# Duckweed Species Genotyping and Interspecific Hybrid Discovery by Tubulin-Based Polymorphism Fingerprinting

**DOI:** 10.3389/fpls.2021.625670

**Published:** 2021-03-08

**Authors:** Luca Braglia, Massimiliano Lauria, Klaus J. Appenroth, Manuela Bog, Diego Breviario, Aldo Grasso, Floriana Gavazzi, Laura Morello

**Affiliations:** ^1^Institute of Agricultural Biology and Biotechnology, National Research Council, Milan, Italy; ^2^Institute of Plant Physiology, Friedrich Schiller University Jena, Jena, Germany; ^3^Institute of Botany and Landscape Ecology, University of Greifswald, Greifswald, Germany

**Keywords:** duckweeds, genotyping, interspecific hybrids, tubulin-based polymorphism, *Lemna japonica*, DNA barcoding, interspecific polymorphism

## Abstract

Duckweeds (Lemnaceae) are the smallest and fastest-growing angiosperms. This feature, together with high starch production and good nutritional properties, makes them suitable for several applications, including wastewater treatment, bioenergy production, or feed and food supplement. Due to their reduced morphology and great similarity between diverse species, taxonomic identification of duckweeds is a challenging issue even for experts. Among molecular genotyping methods, DNA barcoding is the most useful tool for species identification without a need for cluster analysis. The combination of two plastid barcoding loci is now considered the gold standard for duckweed classification. However, not all species can be defined with confidence by these markers, and a fast identification method able to solve doubtful cases is missing. Here we show the potential of tubulin-based polymorphism (TBP), a molecular marker based on the intron length polymorphisms of β-tubulin loci, in the genomic profiling of the genera *Spirodela*, *Landoltia*, and *Lemna*. Ninety-four clones were analyzed, including at least two representatives of each species of the three genera, with a special focus on the very heterogeneous species *Lemna minor*. We showed that a single PCR amplification with universal primers, followed by agarose gel analysis, was able to provide distinctive fingerprinting profiles for 10 out of 15 species. Cluster analysis of capillary electrophoresis–TBP data provided good separation for the remaining species, although the relationship between *L. minor* and *Lemna japonica* was not fully resolved. However, an accurate comparison of TBP profiles provided evidence for the unexpected existence of intraspecific hybrids between *Lemna turionifera* and *L. minor*, as further confirmed by amplified fragment length polymorphism and sequence analysis of a specific β-tubulin locus. Such hybrids could possibly correspond to *L. japonica*, as originally suggested by E. Landolt. The discovery of interspecific hybrids opens a new perspective to understand the speciation mechanisms in the family of duckweeds.

## Introduction

Duckweeds (Lemnaceae) are the smallest and fastest-growing angiosperms. This feature, together with high starch production and good nutritional properties, makes them suitable for several applications, including wastewater treatment (Körner et al., 2003), bioenergy production (Cui and Cheng, 2015), or feed and food supplement (Appenroth et al., 2017). Duckweeds are a rather small but complex angiosperm group, including 36 species across five genera, monophyletic with Araceae (Nauheimer et al., 2012). These plants are spread in any continent, except the Antarctic, where they populate slow-moving or still freshwaters. Their extremely simplified body plan is the result of a neotenic adaptation to their freely floating aquatic lifestyle. A progressive reduction in size and complexity is observed across their evolution. The morphologically more differentiated *Spirodela*, also called giant duckweed, is endowed with larger fronds and several roots, whereas the more recently diverged genera *Wolffia* and *Wolffiella* show miniaturized, rootless fronds with a size of up to 1-mm small (Landolt, 1986; Bog et al., 2020a).

Although many duckweed species can rarely flower in nature, asexual propagation through budding from mother fronds is the usual proliferation mechanism.

Genetic variability solely provided by the slow accumulation of somatic mutations without recombination could hardly explain the evolutionary radiation of duckweeds, whose underlying speciation mechanisms are then of particular interest. A clear definition of duckweed taxonomy and the availability of effective tools for species delimitation are fundamental to this purpose. However, duckweed’s reduced morphology, together with the rare occurrence of flower and seed formation, and the similarity of many morphological characters (Cleland, 1985; Landolt and Kandeler, 1987) limit the number of morphological traits useful for taxonomy. This fact, together with the high intraspecific phenotypic variability observable under different environmental conditions, often makes unequivocal identification of some species a challenging issue even for expert taxonomists (Bog et al., 2020a).

Since the comprehensive work of Les et al. (2002), who first used plastid sequences as DNA markers, later followed by nuclear markers (Tippery et al., 2015), molecular taxonomic analysis was used to support the internal structure of the duckweed family, which was previously based only on morphological and biochemical data (Les et al., 1997; reviewed in Sree et al., 2016 and Bog et al., 2019). Different genotyping methods have been applied to the resolution of the phylogenetic relationships among duckweeds, gradually shaping the currently accepted evolutionary tree and allowing the identification of synonymous species. This refining work is still ongoing, as demonstrated by the most recent taxonomic revision that reduced the number of duckweed species from 37 to 36, by synonymizing *Lemna valdiviana* Phil. and *Lemna yungensis* Landolt (Bog et al., 2020b,c).

The work of Wang et al. (2010) proposed the plastid intergenic spacer *atpF-atpH* as the most promising barcoding marker for species-level identification in duckweeds by analyzing 31 species. By extending this analysis to a larger number of representative clones of all duckweed species, Borisjuk et al. (2015) proposed the use of this marker in combination with a second plastid spacer sequence, *psbK-psbI*, for higher resolution. A Bayesian tree-based classification was able to discriminate most, but not all, species by the combination of the two markers. Faster species recognition based on a PCR sequence–Basic Local Alignment Search Tool (BLAST) protocol allowed the unambiguous identification of 25 out of 37 (now 36) duckweed species, while for five species the need for further sequence data from more clones was highlighted in order to establish the effectiveness of the protocol. For instance, the high degree of nucleotide identity found among the spacer sequences of the two sister species *Lemna japonica* Landolt and *Lemna minor* L., resulting in the lack of difference between inter- and intraspecific genetic distances (barcoding gap), makes it unfeasible to distinguish one from the another by this method.

Fingerprinting by amplified fragment length polymorphism (AFLP) has also been successfully applied to duckweed taxonomy (Bog et al., 2019), allowing a good delineation of all *Lemna* species by cluster analysis, with the exception of *Lemna gibba* L. This species, representing a large subset of the analyzed clones, appeared split into four non-monophyletic clades (Bog et al., 2010), raising doubts about the classification of some of the clones.

Although different molecular markers support the current duckweed taxonomy and sequencing of one or two DNA barcoding regions seems the simplest method for the identification of most duckweed species, the previously mentioned limits highlight the lack of a reliable and fast tool to characterize newly identified clones. The unequivocal taxonomical identification of the different species and clones is strongly required for utilizing duckweeds in several applications, including wastewater treatment (Körner et al., 2003; Ceschin et al., 2020), bioenergy production (Cui and Cheng, 2015), feed and food supplement (Kaplan et al., 2019), and management of stock collections.

A correct identification becomes fundamental also for the management and conservation of native duckweeds, such as *L. minor*, which is more and more often replaced in nature by a similar invasive alien *Lemna minuta* Kunth (Ceschin et al., 2016a).

Tubulin-based polymorphism (TBP) is a PCR-based, multilocus, codominant nuclear marker, targeting β-tubulin introns (Bardini et al., 2004; Gavazzi et al., 2012), which has been widely tested for plant genotyping at the level of species and lower ranks, e.g., cultivar and ecotypes, in many different genera such as *Vitis* (Gavazzi et al., 2016), *Olea* (Braglia et al., 2017), *Passiflora* (Braglia et al., 2014), and *Triticum* (Silletti et al., 2019). Similar to AFLP and simple sequence repeats (SSR), polymorphic profiles are generated by amplicon length variation across clones, but differently to these markers, TBP is both fully transferable across species and a non-random marker, with defined genomic target loci that correspond to the members of the β-tubulin multigene family. This strategy is possible because all members of this gene family, without known exceptions, share a unique exon–intron organization across land plants, from bryophytes to angiosperms, with two introns of variable length at fixed positions (Breviario et al., 2013). Due to the strong structural constraints that limit amino acid variation in tubulin, nucleotide regions highly conserved across all plant species are found in the flanking regions of both introns, allowing the design of two degenerated universal primer pairs that can potentially cover all Embryophyta species.

The goal of this work was to explore the potential of the TBP marker, not yet tested on the duckweed family, as a possible reliable tool for discrimination among duckweed species and clones. Species of the genera *Spirodela*, *Landoltia*, and *Lemna* were selected for this first test. TBP was shown to provide distinctive fingerprinting profiles at the species and, in some cases, the clone level. It was also useful to unravel relationships between some closely related *Lemna* species, relying on interspecific hybridization.

## Materials and Methods

### Plant Material and DNA Extraction

The plant material consisted of 94 duckweed clones, distributed across three genera and 15 species, coming from stock collections (Table 1). The clones are named with the Landolt four-digit code or from the owner of the collection. At least two clones of each species and six *Lemna* clones not yet assigned to a species were selected. The duckweed plants were cultured aseptically on Schenk and Hildebrandt agar medium, supplied with 0.2% sucrose, in a growth chamber at 25°C, with a light-and-dark regime of 16 and 8 h, respectively (photon flux of 31–34 μmol m^–2^ s^–^). Total DNA was individually extracted from 100 mg of duckweed fronds. DNA isolation was performed according to the standard protocol of the DNeasy Plant Mini Kit (Qiagen, Valencia, CA, United States). Fresh tissue was ground in a 2-ml tube in extraction buffer, with three 3-mm stainless steel beads, using a TissueLyser II apparatus (Qiagen, Hilden, Germany) at a frequency of 30 Hz for 1 min. DNA quality and amount were determined by UV absorbance with the Nanodrop 2000C (Thermo Fisher Scientific, Inc., Waltham, MA, United States), and DNA was stored at −20°C until use.

**TABLE 1:** List of tested clones, with indication of the original classification in the collection and the geographical origin.

**Clone ID**	**Collection**	**Species**	**Continent/region**	**Country**	**State/city**
LM0001	CNR-IBBA	*Spirodela polyrhiza* (L.) Schleid.	Asia	Thailand	Phuket
LM0002	CNR-IBBA	*Spirodela polyrhiza*	Europe	Russia	Uva, Udmurtia
LM0003	CNR-IBBA	*Spirodela polyrhiza*	Europe	Russia	Lugovaya, Lobnya, Moscow region
9509	Jena Univ.	*Spirodela polyrhiza*	Europe	Germany	Lotschen, Stadtroda
9500	Jena Univ.	*Spirodela polyrhiza*	Europe	Germany	Jena, Porstendorf
7498	Jena Univ.	*Spirodela polyrhiza*	North America	United States	North Carolina, Durham Co., Durham
7657	Jena Univ.	*Spirodela polyrhiza*	North America	Mexico	Veracruz, Coatzacoalcos
8483	Jena Univ.	*Spirodela polyrhiza*	North America	United States	North Carolina, Dare Co., Nags Head Woods
7450	Jena Univ.	*Spirodela intermedia* W. Koch	Asia	India	Delhi, Botanical Garden
8410	Jena Univ.	*Spirodela intermedia*	South America	Panama	Panama City
9354	Jena Univ.	*Landoltia punctata* (G. Mey.) Les & D.J. Crawford	Europe	Switzerland	Castel San Pietro, Canton of Ticino
9234	Jena Univ.	*Landoltia punctata*	South America	Ecuador	Esmerelda, Viche
7260	Jena Univ.	*Landoltia punctata*	Australia	Victoria	Portland, Tyrendarra
9637	Jena Univ.	*Landoltia punctata*	Australia	New South Wales	Armidale
7449	Jena Univ.	*Landoltia punctata*	Asia	India	Delhi
9604	Jena Univ.	*Landoltia punctata*	Asia	China	Kunming
7760	Jena Univ.	*Landoltia punctata*	Australia	South Australia	Mt. Gambier, Caroline Sinkhole
9245	Jena Univ.	*Landoltia punctata*	Asia	Vietnam	U Minh, Kien Giang
9526	Jena Univ.	*Lemna aequinoctialis* Welw.	Asia	India	Telangana
9925°	Jena Univ.	*Lemna aequinoctialis*	Asia	Bangladesh	Dhaka
9593	Jena Univ.	*Lemna aequinoctialis*	Asia	India	Assam, Guwahati
7842	Jena Univ.	*Lemna disperma* Hegelm.	Australia	South Australia	Mt. Gambier
7269	Jena Univ.	*Lemna disperma*	Australia	Tasmania	Sorell
7245	Jena Univ.	*Lemna gibba* L.	Africa	South Africa	Cape, Stellenbosch, Jonkershoek
7742a	Jena Univ.	*Lemna gibba*	Europe	Italy	Sicily
8124	Jena Univ.	*Lemna gibba*	North America	United States	Arizona, Pima Co., Arivaca
9562	Jena Univ.	*Lemna gibba*	Europe	Italy	Lake Trasimeno, Perugia Province
9481	Jena Univ.	*Lemna gibba*	Europe	Denmark	Mon
7796	Jena Univ.	*Lemna gibba*	Europe	Italy	Sicily, Province of Catania
9257	Jena Univ.	*Lemna japonica* Landolt	Europe	Finland	South Häme, Lake Vesijärvi
8695	Jena Univ.	*Lemna japonica*	Asia	Japan	Kyoto, Yodo
9017	Jena Univ.	*Lemna japonica*	Asia	Japan	Kyushu, Usa City
9252	Jena Univ.	*Lemna japonica*	Europe	Finland	Uusimaa, Haltiala
8693	Jena Univ.	*Lemna japonica*	Asia	Japan	Kyoto, Yodo
9283	Jena Univ.	*Lemna japonica*	Asia	China	Wuhan, Hubei
8676	Jena Univ.	*Lemna minor* L.	Asia	India	Kashmir
9436b	Jena Univ.	*Lemna minor*	Europe	Albania	Southern part
9536	Jena Univ.	*Lemna minor*	Europe	Germany	Berlin, Schildow Nr. 3
9533	Jena Univ.	*Lemna minor*	Europe	Macedonia	Krusje
9355	Jena Univ.	*Lemna minor*	Europe	Germany	Thuringia, Lotschen/Jena
9440	Jena Univ.	*Lemna minor*	Europe	Germany	Thuringia, Eisenach
8389	Jena Univ.	*Lemna minor*	Africa	South Africa	Transval
8625	Jena Univ.	*Lemna minor*	Europe	Norway	Oslo-Honefoss
7766	Jena Univ.	*Lemna minor*	Pacific	New Zealand	Southern Island
8744	Jena Univ.	*Lemna minor*	Europe	Albania	Lezha
8627	Jena Univ.	*Lemna minor*	Europe	Denmark	Sjaelland, Copenhagen, Slangerup
LM0004	CNR-IBBA	*Lemna minor*	Europe	Russia	Lobnya, Moscow region
LM0005	CNR-IBBA	*Lemna minor*	Europe	Russia	Lugovaya, Lobnya, Moscow region
LM0006	CNR-IBBA	*Lemna minor*	Europe	Russia	Vidnoe, Moscow region
LM0007	CNR-IBBA	*Lemna minor*	Asia	Russia	Altai Krai, Barnaul, Zmeinogorsky track, 49
LM0008	CNR-IBBA	*Lemna minor*	Asia	Russia	Altai Republic, Turochaksky District, Lake Teletskoye
7194	Jena Univ.	*Lemna minor*	Africa	Uganda	Masaka
8623	Jena Univ.	*Lemna minor*	Europe	Denmark	Ijland Alborg
9495	Jena Univ.	*Lemna minor*	Europe	Norway	Stavanger
9437	Jena Univ.	*Lemna minor*	Europe	Italy	Südtirol, St. Joseph (Appenroth SO 5)
8292	Jena Univ.	*Lemna minor*	Asia	Iran	Mazandaran, Ramsar, Ghassem Abbath
7210	Jena Univ.	*Lemna minor*	Africa	South Africa	Cape, Grahamstown, “Rockeby Park”
7753	Jena Univ.	*Lemna minor*	Africa	Ethiopia	Hara, Semien, Djinbar-Wans
5500	Jena Univ.	*Lemna minor*	Europe	Ireland	County Cork, Blarney
7022	Jena Univ.	*Lemna minor*	Europe	Spain	Andalusia, Cordoba
KJA0017	Jena Univ.	*Lemna minor*	Europe	Germany	Naumburg, Cathedrale
9441	Jena Univ.	*Lemna minor*	Europe	Germany	Marburg St
9580	Jena Univ.	*Lemna minor*	Europe	Greece	Lithopos, Kerkini lake
KJA0013	Jena Univ.	*Lemna minor*	Europe	Albania	Blue Eye Area, Village Muzine
KJA0016	Jena Univ.	*Lemna minor*	Europe	Germany	Naumburg, Cathedrale
9414	Jena Univ.	*Lemna minuta* Kunth	Europe	Italy	Emilia, Po river
7724	Jena Univ.	*Lemna minuta*	Europe	France	Biarritz, Lac Marion
7612	Jena Univ.	*Lemna minuta*	South America	Peru	Cuzco, San Geronimo
9260	Jena Univ.	*Lemna minuta*	Europe	Italy	Sicily, Catania, Botanical Garden
9342	Jena Univ.	*Lemna obscura* (Austin) Daubs	South America	Venezuela	Lake Maracaibo
7325	Jena Univ.	*Lemna obscura*	Pacific	United States	Hawaii, Oahu, Pearl City
7133	Jena Univ.	*Lemna obscura*	North America	United States	Louisiana, Orleans Par.
8539	Jena Univ.	*Lemna perpusilla* Torr.	North America	United States	Virginia, Norfolk Co., Chesapeake
BOG0007	Greifswald Univ.	*Lemna perpusilla*	North America	United States	Georgia, Charlton Co., Okefenokee, Chesser Island, 20 km SW of Folkston
BOG0001	Greifswald Univ.	*Lemna perpusilla*	North America	United States	Florida, Martin Co., Allapatha flats, 12 km NNW of Indiantown
8473	Jena Univ.	*Lemna perpusilla*	North America	United States	North Carolina, Johnston Co., Gees Cross Road
9020	Jena Univ.	*Lemna tenera* Kurz	Australia	Northern Territories	Condorl Water Hole
9024	Jena Univ.	*Lemna tenera*	Australia	Northern Territories	Nancar Billabong
9529	Jena Univ.	*Lemna trisulca* L.	Europe	Germany	Jena
5555	Jena Univ.	*Lemna trisulca*	Europe	Germany	Thuringia, Hainich National Park
9434	Jena Univ.	*Lemna turionifera* Landolt	Asia	Russia	Baikal lake
BOG0006	Greifswald Univ.	*Lemna turionifera*	Asia	Russia	Baikal Lake, Irkutsk Oblast
9530	Jena Univ.	*Lemna turionifera*	Europe	Albania	Lake Prespa
9229	Jena Univ.	*Lemna valdiviana* Phil.	South America	Ecuador	Pichincha, Rio Chiche
8685	Jena Univ.	*Lemna valdiviana*	South America	Chile	Cautin, Temuco
8831	Jena Univ.	*Lemna valdiviana*	South America	Argentina	Formosa, Laguna Blanca
9208	Jena Univ.	*Lemna valdiviana*^a^	South America	Bolivia	La Paz, NNE of Sacramento
9207	Jena Univ.	*Lemna valdiviana*^a^	South America	Bolivia	La Paz, NNE of Sacramento
9614	Jena Univ.	*Lemna* spp.	Europe	Poland	Topilo, reservate, east of railway track
9583	Jena Univ.	*Lemna* spp.	Europe	Poland	Topilo
9619	Jena Univ.	*Lemna* spp.	Europe	Albania	Water purification plant, Pogradeci
LM0009	CNR-IBBA	*Lemna* spp.	Europe	Italy	Po river
LM0010	CNR-IBBA	*Lemna* spp.	Europe	Italy	Fontana dell’Olmo, Pitigliano, Grosseto province
LM0011	CNR-IBBA	*Lemna* spp.	Europe	Russia	Serebryano-Vinogradny Pond, Moscow region

*^a^Formerly yungensis.*

*Univ., University.*

### TBP Amplification, Capillary Electrophoresis, and Data Analysis

Thirty nanograms of genomic DNA was used as a template for each TBP 1st and 2nd intron amplifications. The PCR reaction was performed in 30 μl according to Braglia et al. (2020), and control reactions without DNA template were included in any experiment. Each DNA sample was tested twice. The FAM-labeled amplicons were visualized on 2% agarose gel to check for amplification signal intensity and opportunely diluted in double-distilled water. The capillary electrophoresis–TBP (CE-TBP) separation samples were prepared using 2 μl of each diluted PCR product. Capillary electrophoresis and data collection were performed according to the parameters defined by Braglia et al. (2020) using the Gene Mapper Software v. 5.0 tools (Thermo Fisher Scientific, Inc., Waltham, MA, United States) independently for the two intron regions (1st and 2nd). Raw numerical data concerning the size (in base pairs) and the height (in relative florescence units, RFUs) of each CE-TBP profile peak were converted into Microsoft Office Excel files.

The comparison of the CE-TBP profiles of the analyzed clones and the sorting of the numerical data were performed according to the peak size. The peak size was considered as a marker, and its presence/absence was scored in a binary matrix (1/0, respectively). Both TBP 1st and 2nd intron were scored. The genetic dissimilarity values, among the analyzed genotypes, were estimated using the open source software R v. 3.6.2 (R Core Team, 2019), as implemented in the package “ecodist” v. 2.0.5 (Goslee and Urban, 2007) according to Jaccard’s index for binary data. Dendrograms were computed by the neighbor joining (NJ) algorithm using the R package “ape” v. 5.3 (Paradis and Schliep, 2018). The statistical confidence of a particular group of clones within the obtained tree was evaluated by a bootstrap test with 1,000 replicates.

To measure the correlation between the genetic distance matrices estimated by scoring the markers from the TBP 1st and 2nd intron regions, the Mantel test (Mantel, 1967) was carried out with the batch file of the NTSYS-pc2.10e software (Rohlf, 1998). The same test was also used to test TBP repeatability by comparison of the genetic distance matrices estimated for two independent TBP 1st intron amplifications of 32 duckweed clones selected within the *L. minor* group.

### Single Tubulin Gene Amplification

Gene-specific primer pairs annealing to the first intron–exon borders of the two homoeologous β-tubulin loci *TUBB* 11-1 and *TUBB* 23-1 were manually designed on the alignment of the two sequences. Two base pair degenerations were included at polymorphic positions. Primers (forward I-Fw_11-23_1 5′-TTC AGG GTA TGC GAT CTA TTC-3′ and reverse I-Rv_11-23_1 5′-GGA ATC CTG CAM KTA AAT GAY G-3′) were used to perform endpoint PCR amplifications by a standard protocol, using 10 ng of gDNA template in a total reaction volume of 20 μl with 2X Taq DNA Polymerase Master Mix (VWR International srl, Milan, Italy). Four microliters of the PCR products was analyzed on 2% (w/v) agarose gel stained with Atlas ClearSight DNA stain (Bioatlas, OÜ, Tartu, Estonia). After PCR purification or band cutting from gel, if two amplicons were present, the PCR products of selected clones were sequenced on both strands by an external service (Microsynth, Balgach, Switzerland) using amplification primers. The obtained sequences were aligned with the corresponding *L. minor* genome sequences by the Align X tool of the Vector NTI Advance 11.5 suite (Thermo Fisher Scientific, Inc., Waltham, MA, United States).

### *In silico* β-Tubulin Gene Sequence Analysis

The *L. minor* 8627 and the *L. minor* 5500 genome assemblies were retrieved from https://www.lemna.org and https://genomevolution.org/r/ik6h, respectively, and used for a stand-alone BLAST search, using a *Spirodela polyrhiza* β-tubulin gene sequence as a query. Then, on the basis of BLAST hit coordinates, β-tubulin sequences were extracted from the corresponding genome by using SAMtools (Li et al., 2009).

The whole genomic regions encompassing the open reading frame, including introns, were aligned by ClustalW2 software included in the MEGA X version 10.1.7 suite (Kumar et al., 2018). The evolutionary distances were computed using the p-distance method (Nei and Kumar, 2000), and all sequence positions containing gaps and missing data were eliminated (complete deletion option). The phylogenetic analysis was inferred through the UPGMA method (Sneath and Sokal, 1973), and the percentage of replicate trees in which the associated taxa clustered together was estimated by bootstrap test (1,000 replicates) (Felsenstein, 1985). DNA-seq reads of the *L. minor* 5500 clone, SRR2879345 and SRR2879346, were retrieved from the Genbank database^1^. For adapter trimming and quality control, Trim Galore wrapper script (version 0.6.6)^2^ was used. Then, the resulting quality-filtered reads were used for mapping against the *L. minor* 8627 genome with Bowtie2 (version 2.3.4.1; Langmead and Salzberg, 2012) using default parameters.

### DNA Barcoding and Sequence Analysis

Twenty nanograms of total genomic DNA was used for amplification of the *atpF*-*atpH* spacer using 0.5 μM of each primer (Fw_Bar_atpF_atpH: ACTCGCACACACTCCCTTTCC; Rv_Bar_atpF_atpH: GCT TTTATGGAAGCTTTAACAAT) (Wang et al., 2010) in a final volume of 20 μl, with 1 unit Platinum Taq DNA Polymerase (Invitrogen, Thermo Fisher Scientific, Inc., Waltham, MA, United States). PCR products were checked on agarose gel and then purified using the QIAquick PCR Purification Kit according to the manufacturer’s instructions (Qiagen, Hilden, Germany). The amplified products were forward- and reverse-sequenced (Microsynth, Balgach, Switzerland). The NCBI BLASTn analysis of resulting contigs was performed for clone identification by best match analysis (see text footnote 1).

### AFLP Analysis

A selection of clones of the *L. minor* group, including the nine used for sequencing of *TUBB* 11-23-1 introns, was analyzed by AFLP fingerprinting using a modification of a previous protocol (Lauria et al., 2004). Briefly, 100 ng of total genomic DNA was digested using the two restriction enzymes *Eco*RI and *Mse*I, following ligation of proper adapters to the restricted DNA fragments. The ligation products, after enzyme inactivation (68°C for 15 min), were diluted threefold and used as a template for pre-selective amplification with the primer pair *Eco*RI −0/*Mse*I + 2. Then, the pre-amplification products were diluted 10-fold, and 3 μl was used as a template for selective amplification with 10 *Eco*RI + 3/*Mse*I + 4 selective primer combinations (PC). The *Eco*RI selective amplification primer was labeled with 6-FAM fluorescent dye at the 5′ end to allow fragment analysis by capillary electrophoresis on a 3500 Genetic Analyzer (Thermo Fisher Scientific, Inc., Waltham, MA, United States). For each clone, two independent AFLP restriction–ligation reactions were performed and tested with the primer combinations PC48 and PC51; once reproducible, the two restriction–ligation reactions of each sample were pooled and used for further analysis. The adaptor and primer sequences used in this study are reported in Supplementary Table 1.

The CE reaction was prepared in a volume of 20 μl by adding 2 μl of the amplified DNA, 0.22 μl of GeneScanTM 500LIZ^TM^ dye size standard (Thermo Fisher Scientific, Inc., Waltham, MA, United States), and 17.78 μl of Hi-DiTM Formamide (Thermo Fisher Scientific, Inc., Waltham, MA, United States). Gene Mapper Software v. 5.0 (Thermo Fisher Scientific, Inc., Waltham, MA, United States) elaborates and processes the data, allowing the sizing and the release of the AFLP peak pherogram output. We used the size range of 50 to 450 base pairs for scoring of all primer combinations. The lower signal threshold for peak detection was set at 150 RFU. The peak size (base pairs) and height (RFUs) of each electropherogram were converted into a Microsoft Office Excel file, and all the AFLP profiles were aligned according to the peak size. A binary matrix was generated for each PC by scoring for the presence/absence of the markers (1/0, respectively).

As measures of genetic divergence, the number of total markers and the fixed private markers in each group of species were calculated using FAMD v.1.31 software (Schlüter and Harris, 2006). The pairwise genetic distances (GDs) were estimated based on the Dice’s similarity coefficient by Past3 software (v3.25) for Windows (Hammer et al., 2001). To obtain a more detailed view of the distribution of genetic variation within and between different groups of clones, mean GD among clones belonging to the same and/or different groups was also calculated.

## Results

### Species Recognition at a Glance

To test the power of the TBP method for duckweed genotyping, we focused on the genera *Spirodela*, *Landoltia*, and *Lemna* by analyzing a large set of clones (Table 1) that are representative of all 15 species of these genera.

**FIGURE 1:**
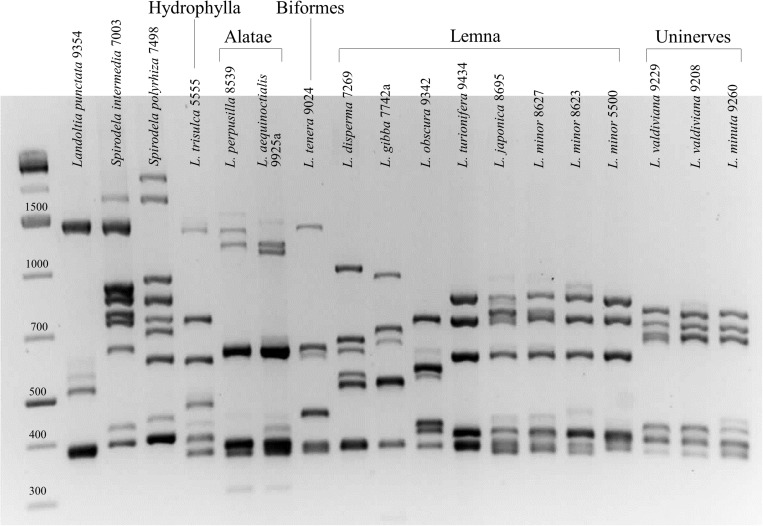
First intron tubulin-based polymorphism fingerprinting of representative clones of all 15 species of the genera *Spirodela*, *Landoltia*, and *Lemna*.

Both the 1st and the 2nd intronic β-tubulin target regions were amplified with 100% success rate. In the first instance, PCR products were analyzed by agarose gel electrophoresis, revealing distinctive band patterns for different species already at a glance. Figure 1 shows the comparison of the first intron fingerprinting of representative clones for each species.

The number of discrete amplified bands in each DNA sample ranged from four [*Landoltia punctata* (G. Mey.) Les & D. J. Crawford] to nine [*S. polyrhiza* (L.) Schleid. and *Spirodela intermedia* W. Koch] for the first intron and from three to five for the second intron (not shown). Since each β-tubulin gene is expected to have two introns, the discordant number of bands is likely due to co-migration of similar-length amplicons. Distinctive species-specific profiles were clearly visible for 10 out of 15 of the investigated species, providing easy identification also in those cases in which the morphological criteria are not always straightforward. The two *Spirodela* species, *S. polyrhiza* and *S. intermedia*, were readily distinguished from one another and from *L. punctata* until, in 1999, considered a congeneric under the names *S. oligorrhiza* (Kurz) Hegelm. or *S. punctata* (Les and Crawford, 1999). The last species showed a very distinct profile on agarose gel, with fewer amplicons (Figure 1). *L. gibba* was clearly distinct from *L. minor* for which it is easily mistaken (Landolt, 1975). In the section Alatae, the two sister species *Lemna perpusilla* Torr. and *Lemna aequinoctialis* Welw. showed a similar pattern but can be distinguished by the size of their high-molecular-weight amplicon doublet. Two notable exceptions were represented by two groups of species showing hardly distinguishable band patterns. One was the section Uninerves, in which the sister species *L. minuta* and *L. valdiviana* (Crawford et al., 1996) showed nearly identical band patterns, also shared by those clones formerly classified as *L. yungensis* (clone 9208 in Figure 1), data that is consistent with the fact that the last two species are now considered as synonymous (Bog et al., 2020c). The second group of species showing highly similar profiles included *L. minor* and *L. japonica*, also known to be closely related and sharing most morphological traits (Landolt, 1986). More unexpected was their similarity with *Lemna turionifera* Landolt, which is more distantly related according to the most recent phylogenetic tree based on both nuclear and plastid nucleotide sequences (Tippery and Les, 2020). The band patterns of three different *L. minor* clones are shown in Figure 1, accounting for their similarity with the two other species. The relationships among *L. minor*, *L. japonica*, and *L. turionifera*, better investigated by capillary electrophoresis fragment separation, are described below in a specific chapter.

### Intraspecific Polymorphism by TBP

**FIGURE 2:**
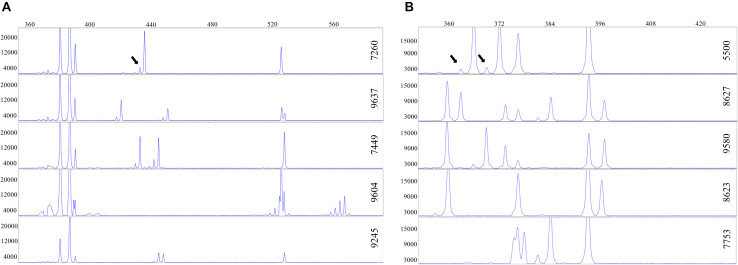
Intraspecific polymorphism in duckweeds, highlighted by capillary electrophoresis. A portion of the electropherogram is shown. The vertical and horizontal axes reflect peak intensity (relative fluorescence units) and peak size (base pairs), respectively. Arrows point to stutter peaks. **(A)** 1st intron tubulin-based polymorphism (TBP) profiles of five different *Landoltia punctata* clones. **(B)** 1st intron TBP profiles of five *Lemna minor* clones.

The TBP-amplified DNA of all the clones was also analyzed by capillary electrophoresis that provides electropherogram peak profiles with single nucleotide resolution (CE-TBP, Figures 2A,B).

This allowed to uncover a certain range of intraspecific variability, particularly in the 1st intron, with small size differences ranging from one to few nucleotides. Such polymorphism likely represents allelic InDel intron variants as also found among cultivars of crop plants such as wheat or grape (Gavazzi et al., 2016; Silletti et al., 2019). The degree of intraspecific variability was, however, different between species. In *L. punctata* five different allele combinations were detected among eight clones investigated (Figure 2A), while in *S. polyrhiza*, intraspecific variability among eight analyzed clones of different geographic origin was sufficient to provide unique profiles for each clone. The two *S. intermedia* clones showed identical TBP profiles. The numerical data for these three species are available in Supplementary Table 2.

The presence of high-level intraspecific polymorphism in the genus *Lemna* was remarkably evident in *L. minor*, where all the investigated clones showed unique TBP profiles. For instance, Figure 2B shows the 1st intron CE-TBP profiles of five different representative *L. minor* clones, three of which (5500, 8623, and 8627) are previously shown in Figure 1. The highly polymorphic fragment region between 350 and 430 bp in Figure 2B, corresponding to the group of bands poorly resolved by the agarose gel in Figure 1, is here well resolved by capillary electrophoresis. Comparing the analyzed clones, both amplicon number and size were different, thus providing an unambiguous identification of each clone. The presence of stutter peaks (minor products that are one to four repeat units shorter than the main allele peak due to polymerase slipping during DNA synthesis) was strongly suggestive of the presence, within target sequences, of SSR contributing to intron length polymorphisms. Although SSR amplification patterns are known to be reproducible (Flores-Rentería and Krohn, 2013), we tested profile repeatability by two independent replicates of the 1st intron TBP amplification and data analysis of 32 clones of the *L. minor* group in order to exclude the possibility that such PCR artifacts might interfere with an unambiguous identification of clones. The correlation coefficient between the matrices generated by the scored marker for the two sample batches, estimated by the Mantel test, was *r* = 0.95, indicating high repeatability.

In most other *Lemna* species, intraspecific variation was detected by TBP, although to a different extent: for example, only two alternative TBP fingerprints were identified in four *L. minuta* clones. In some cases, the number of investigated clones was too low to address intraspecific variability.

### Cluster Analysis of the Genus *Lemna*

All 76 clones representative of the 12 *Lemna* species, including six newly isolated *Lemna* clones not classified before by morphological or molecular markers (*Lemna* spp. in Table 1), were included in the cluster analysis to test TBP for its ability to assign clones to their respective species. *S. polyrhiza* clone 7498 was used as the outgroup species. The size range of the scored CE-TBP peaks was between 253 and 1,292 bp for the 1st intron and between 230 and 947 for the 2nd intron region, revealing 162 TBP polymorphic markers across the considered clones (104 and 58 from the 1st and the 2nd intron, respectively). Raw data of a complete set of CE-TBP analysis are provided in Supplementary Table 3. Since the Mantel test revealed a good correlation (*r* = 0.89) between the similarity matrices obtained from the marker scoring of the two TBP intron regions, the combination of the two was used to explore inter- and intraspecific variability.

**FIGURE 3:**
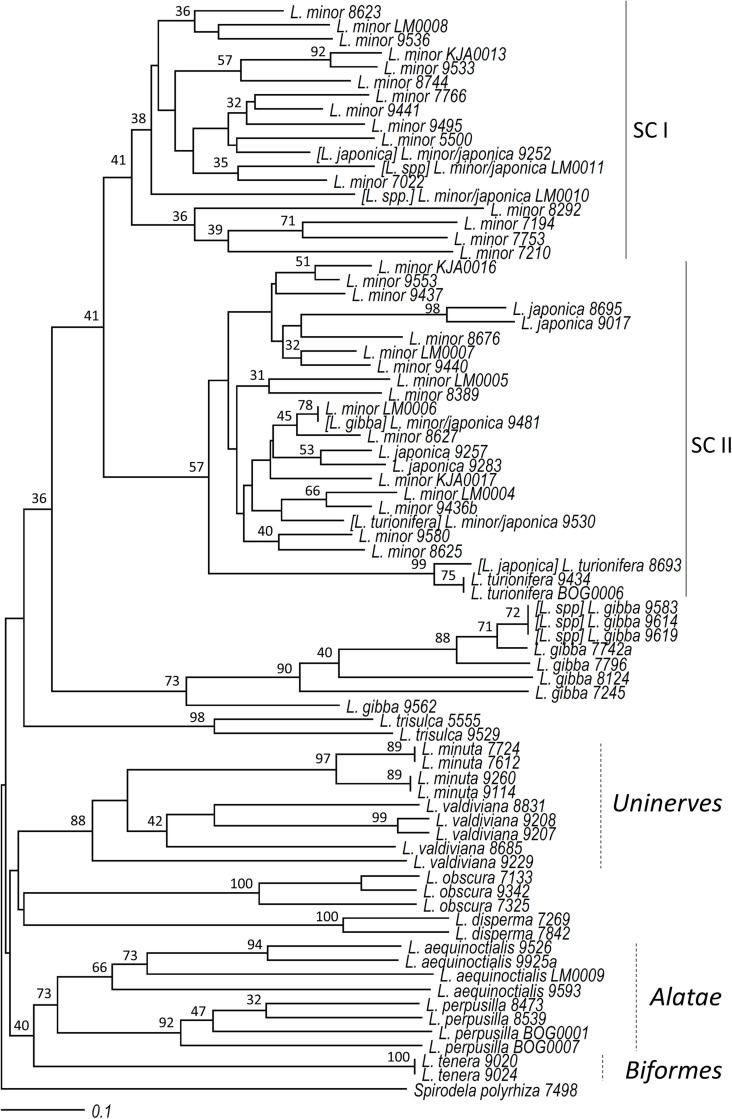
Neighbor joining cluster analysis of clones of the genus *Lemna*. Bootstrap values (≥30) are shown at the nodes of the tree. The names in brackets refer to the original classification in the stock collection, here revised according to tubulin-based polymorphism and *atpF-atpH* DNA barcoding.

Cluster analysis (Figure 3) supported the morphological classification of clones, demonstrating the overall reliability of the TBP approach. Some clones were reclassified or newly classified in this work, and their original classification is given in brackets (see below). A close clustering of clones of the same species was evident, with most species clearly separated. The sub-clusters grouping clones of the *Lemna* sections *Uninerves* and *Alatae* were supported by bootstrap values of 88 and 62%, respectively, while no support was given to the *Lemna* section *Lemna*, probably too divergent for the power of the marker.

Unlike agarose gel analysis, cluster analysis based on CE-TBP also separated *L. minuta* from the sister species *L. valdiviana* (*Lemna* section *Uninerves*), while the separation between clones of *L. valdiviana* and those formerly classified as *L. yungensis* (clones 9207 and 9208) was not supported, in accordance with their recent synonymization. The positioning of clone 9229 (*L. valdiviana*) outside the branch calls for further analysis to be performed on a larger number of clones in order to provide a better resolution of the cluster. The sister species *L. perpusilla* and *L. aequinoctialis* also formed two separated branches with a common origin (62% bootstrap value). Four *L. gibba* clones clustered in a clearly monophyletic group together with three previously unclassified *Lemna* clones 9583, 9614, and 9619. However, another *L. gibba* clone (9481) found an unexpected positioning in the NJ tree with respect to their morphological classification, clustering within the *L. minor*/*L. japonica* group. This largely represented group (42 clones) included clones of four different species: all clones of *L. minor*, *L. japonica*, and *L. turionifera* present in our study, the just mentioned *L. gibba* 9481, and two of the previously unclassified clones, LM0010 and LM0011. While the first three species are known to be related, the presence of this *L. gibba* clone, although unexpected, was consistent with its possible misclassification resulting from a previous AFLP-based cluster analysis which positioned it close to *L. turionifera* (Bog et al., 2010).

The *L. minor* cluster appeared to be clearly split in two major subclusters, indicated as subcluster I (SC I) and subcluster II (SC II) in Figure 3. *Lemna minor*-SC I included only clones classified as *L. minor* by morphology, with the only exception of clone 9252 registered as *L. japonica*. Conversely, SC II included clones of the four mentioned species: *L. minor*, *L. japonica*, *L. turionifera*, and *L. gibba*. While no species separation was evident for *L. japonica*, *L. minor*, and the *L. gibba* clone in this group, a separated and well-supported branch hosted three *L. turionifera* clones. Interestingly, one of these, clone 8693, was classified by E. Landolt originally as *L. japonica* but was later corrected to *L. turionifera* (personal communication to KJA). This change makes this branch homogeneous, although it does not include a fourth *L. turionifera* clone, 9530, which fell outside this branch, in SC II.

### TBP Validation by *atpF-atpH* Barcoding and Morphological Analysis

The heterogeneous composition of *L. minor*-SC II, revealing some inconsistencies between morphological analysis and TBP profiling, called for a necessary validation of the latter. Such a validation was also needed for the six *Lemna* clones that were newly classified by the TBP method (*Lemna* spp. in Table 1 and Figure 3). In addition, 10 other clones of different species, with still missing molecular marker analysis, were included in the validation. We used the barcoding marker *atpF*-*atpH*, which is considered as the gold standard for species identification in duckweeds (Wang et al., 2010; Borisjuk et al., 2015), although ambiguity between the sister species *L. aequinoctialis*/*L. perpusilla*, *L. minor*/*L. japonica*, and *L. minuta*/*L. valdiviana* could not be unequivocally resolved by this marker alone (Borisjuk et al., 2015). The sequence similarity-based identification (best match; cfr. Borisjuk et al., 2015) of the 24 analyzed clones is shown in Table 2. Morphological inspection was also used, in some cases, to support the new classification. For instance, an accurate investigation of distinctive morphological markers such as the position of the papule and the number of nerves spoke in favor of the re-classification as *L. minor* of the only *L. japonica* clone (9252) clustering in *L. minor*-SC I. The TBP classification of clones 9583, 9614, and 9619 as *L. gibba* was clearly confirmed by *atpF-atpH* and by morphology. The sequences of the plastid marker of clones LM0010 and LM0011 showed high homology to both *L. japonica* and *L. minor*, supporting the TBP results. The classification of clone LM0009 as *L. aequinoctialis* was also confirmed by barcoding.

**TABLE 2:** Validation of tubulin-based polymorphism (TBP) data by *atpF-atpH* barcoding or morphological markers.

**Clone**	**Species**		
	**Original classification**	**TBP**	***atpF-atpH***
9434	*L. turionifera*	*L. turionifera*	*L. turionifera*
BOG0006	*L. turionifera*	*L. turionifera*	*L. turionifera*
9530	*L. turionifera*	*L. minor-*SC II	*L. minor/japonica*
9481^a^	*L. gibba*	*L. minor-*SC II	*L. minor/japonica*
8693^a^	*L. japonica*	*L. turionifera*	*L. turionifera*
9252^b^	*L. japonica*	*L. minor-*SC I	*L. minor*
9017	*L. japonica*	*L. minor-*SC II	*L. minor/japonica*
8676	*L. minor*	*L. minor-*SC II	*L. minor/japonica*
8627	*L. minor*	*L. minor-*SC II	*L. minor/japonica*
8744	*L. minor*	*L. minor-*SC I	*L. minor/japonica*
LM0010	*L.* spp.	*L. minor-*SC I	*L. minor/japonica*
LM0011	*L.* spp.	*L. minor-*SC I	*L. minor/japonica*
9614	*L.* spp.	*L. gibba*	*L. gibba*^a^
9583	*L.* spp.	*L. gibba*	*L. gibba*^a^
9619	*L.* spp.	*L. gibba*	*L. gibba*^a^
7245	*L. gibba*	*L. gibba*	*L. gibba*
9020	*L. tenera*	*L. tenera*	*L. tenera*
9024	*L. tenera*	*L. tenera*	*L. tenera*
BOG0001	*L. perpusilla*	*L. perpusilla*	*L. aequinoctialis/perpusilla*
8473	*L. perpusilla*	*L. perpusilla*	*L. aequinoctialis/perpusilla*
9229	*L. valdiviana*	*L. valdiviana*	*L. minuta/valdiviana*
8831	*L. valdiviana*	*L. valdiviana*	*L. minuta/valdiviana*
LM0009	*L.* spp.	*L. aequinoctialis*	*L. aequinoctialis/perpusilla*
7269	*L. disperma*	*L. disperma*	*L. disperma*
9260	*L. minuta*	*L. minuta*	*L. minuta*

*SC I, TBP cluster analysis–subcluster I; SC II, TBP cluster analysis–subcluster II.*

*^a^Confirmed by morphological inspection.*

*^b^Only morphological inspection.*

Validation of TBP results was also obtained for clone 9481, mistaken for *L. gibba*, and for clone 9530, classified as *L. turionifera*, thus resolving major inconsistencies in the *L. minor* group. The classification of these clones according to barcoding is reported in the NJ tree and in Table 1. Finally, clone 8693 was confirmed by barcoding to be *L. turionifera*, in agreement with the previously mentioned last revision by Landolt. The barcoding results were therefore in accordance with TBP analysis in all those cases in which TBP data were discordant with previous morphologic characterization, thus confirming the reliability of the marker. The TBP results were validated for all remaining clones.

### Elucidating the Relationship Between Species in the *L. minor* Clade

Even with the corrections introduced by revised morphological inspections and plastid barcoding analysis, the separation of *L. minor* in two subclusters and the tight intertwining of *L. japonica* and *L. minor* clones in SC II remained open questions. The heterogeneity and complexity of the *L. minor* group has been widely reported (Kandeler, 1975). Large intraspecific variation in genome size, ranging from 323 to 769 Mbp/haploid genome, was found (Wang et al., 2011), suggesting differences in ploidy. For instance, the two fully sequenced *L. minor* clones 8627 (Ernst, 2016) and *L. minor* 5500 (Van Hoeck et al., 2015) have a haploid genome size of 635 and 481, respectively, as estimated by flow cytometry. This difference in size is in accordance with a higher TBP amplicon number found in *L. minor* 8627 if compared to *L. minor* 5500 (12 vs. seven), perhaps explaining the separation of these two clones in the two *L. minor* subclusters in our dendrogram.

**FIGURE 4:**
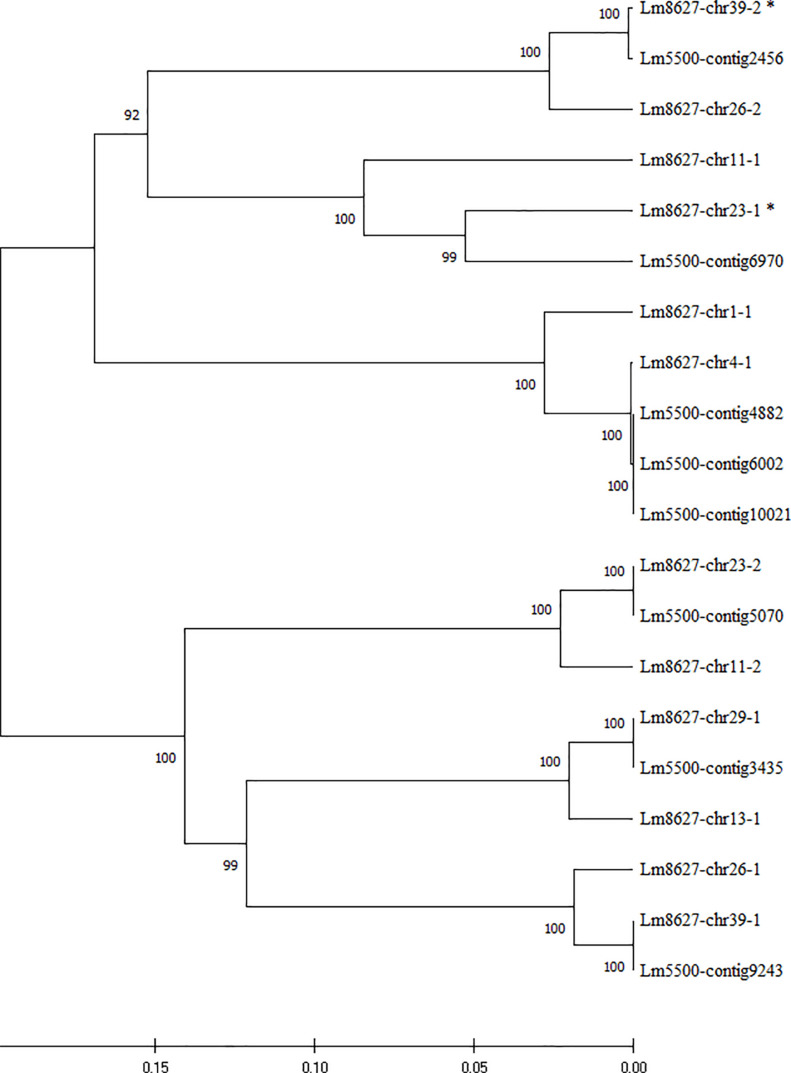
UPGMA tree of the sequence similarity among the β-tubulin genomic sequences of clones *Lemna minor* 8627 and *L. minor* 5500. Genes were named by the contig number in *L. minor* 5500 and by the chromosome location, followed by a serial number, for *L. minor* 8627. Bootstrap values are shown at the nodes of the tree. Asterisks indicate incomplete sequences.

We therefore investigated the composition of the β-tubulin gene family in these two fully sequenced clones. Twelve nearly complete β-tubulin gene sequences of *L. minor* 8627 and eight of *L. minor* 5500, retrieved from genome databases, were aligned. The p-distance tree based on nucleotide sequence similarity is shown in Figure 4. While the β-tubulin gene sequences of clone *L. minor* were associated to contigs, those of *L. minor* 8627 were assigned to eight out of 42 chromosomes due to their physical mapping during the ongoing WGS project, unpublished yet (Ernst et al., 2019). Chromosomes 11, 23, 26, and 39 hosted two independent β-tubulin loci each, whereas chromosomes 1, 4, 13, and 29 had a single β-tubulin gene.

Six gene clusters were identified (Figure 4), each including at least one β-tubulin gene sequence of *L. minor* 5500 and two sequences of *L. minor* 8627. Only in the cluster including sequences *L. minor* 8627-chr 4-1 and *L. minor* 8627-chr 1-1 were three corresponding identical sequences found in *L. minor* 5500 (contigs 4882, 6002, and 10021): this could be due either to a tandem duplication event in this clone or to a sequence misassembly. Interestingly, while a perfect pairwise matching was found between the six genes of *L. minor* 5500 (considering the three identical *L. minor* 5500 sequences as one) and a subset of six β-tubulin genes of *L. minor* 8627 (99–100% pairwise nucleotide identity), the remaining six genes were more distantly related (85.7–96% pairwise nucleotide identity). This finding suggests that two homoeologous genomic subsets are present in clone 8627, likely deriving from the hybridization of two closely related species, with *L. minor* as the donor of one of two subgenomes. This would also explain the positioning of clones 8627 and 5500 in two separate subclusters within the *L. minor* group.

This observation was extended to a genome-wide level by mapping the DNA sequencing data of *L. minor* 5500 against the two chromosome subsets identified in the *L. minor* 8627 genome. The rationale of this analysis was to detect mapping differences between chromosomes that are likely contributed by different parental species. On the basis of Figure 4, chromosomes 1, 11, 13, and 26 were assigned to one parent (subgenome A) and chromosomes 4, 23, 29, and 39 to the second parent, likely *L. minor* (subgenome B). The data presented in Table 3 clearly showed that, on average, the percentage of mapping of set B nearly doubled that of set A: i.e., 18.9 vs. 10.5%.

**TABLE 3:** Percentage of mapping of *L. minor* 5500 DNA sequencing data against the *L. minor* 8627 chromosomes.

	**Chromosome**	**% mapping**	**Total reads analyzed**
Subgenome A	1	9.59	1,000,000
	11	11.72	1,000,000
	13	10.09	1,000,000
	26	10.57	1,000,000
Subgenome B	4	15.47	1,000,000
	23	22.27	1,000,000
	29	18.69	1,000,000
	39	19.31	1,000,000
Subgenome 1	Pseudo_1^a^	29.5	1,000,000
Subgenome 2	Pseudo_2	14.2	1,000,000

*^*a*^The chromosomes of each species were merged, and the resulting file was used for mapping analysis.*

The positioning of *L. turionifera* as a branch in *L. minor*-SC II by cluster analysis, indicative of a high number of CE-TBP common peaks, makes this species the most likely candidate as the donor of the complementary subgenome. In order to verify this possibility, we exploited TBP amplicons as chromosome markers once they were mapped to the chromosomes of *L. minor* 8627. We associated each CE-TBP peak to the corresponding β-tubulin locus by comparison of their size with the *in silico* size prediction. The same was done for clone *L. minor* 5500. As reported in Table 4, an almost precise correspondence was found for all TBP amplicons of both *L. minor* clones. Therefore, each peak could be assigned to the corresponding β-tubulin gene, and all predicted amplicons were identified in the TBP profiles.

**TABLE 4:** Correspondence between capillary electrophoresis–tubulin-based polymorphism profiles and β-tubulin loci of *L. minor* clones 8627 and 5500.

		**TBP 1st**			**TBP 2nd**		
		**Amplicon size**			**Amplicon size**		
	**Gene locus/contig**	**Predicted**	**Detected**	**Intron size**	**Predicted**	**Detected**	**Intron size**
*L. minor* 8627	*TUBB* 1-1	600	598	295	301	299	69
	*TUBB* 4-1	600	598	295	302	301	70
	*TUBB* 13-1	364	361/364^a^	59	304	302	72
	*TUBB* 29-1	388	385	83	304	302	72
	*TUBB* 11-1	756	757	451	302	301	70
	*TUBB* 11-2	376	375	71	503	503	271
	*TUBB* 23-1^b^	729	730	424	302	301	70
	*TUBB* 23-2	379	377	74	501	503	269
	*TUBB* 26-1	401	398	96	296	294	64
	*TUBB* 26-2	742	742	437	948	947	716
	*TUBB* 39-1	397	395	92	297	298	65
	*TUBB* 39-2^b^	824	822	519	827	825	595
*L. minor* 5500	contig6002:4667-6360	600	598	295	302	301	70
	contig4882:16737-18430	600	598	295	302	301	70
	contig10021:2826-4519	600	598	295	302	301	70
	contig5070:18395-20056rw	379	377	74	501	503	269
	contig9243:5991-7483rw	397	395	92	297	298	65
	contig6970:2575-4405rw	729	730	424	302	301	70
	contig2456:16516-18911rw	824	822	519	827	825	595
	contig3435:26387-27804	376	367/373^a^	71	304	302	72

*Gene names refer to the corresponding contig or chromosome number, followed by a serial number.*

*^*a*^Allelic variants due to CTT repeats.*

*^b^Incomplete sequence—the third exon is missing.*

**FIGURE 5:**
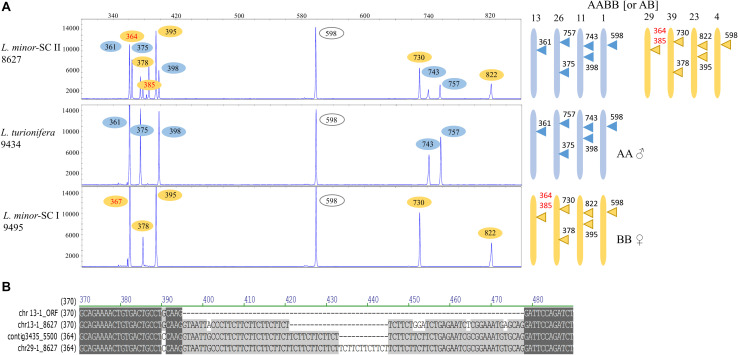
Reconstruction of the possible origin of *Lemna minor* 8627 by hybridization of *Lemna turionifera* and L. minor. **(A)** The 1st intron tubulin-based polymorphism (TBP) profiles of *L. minor* 8627 (SC II), *L. turionifera* 9434, and *L. minor* 9495 (SC I) are shown. The contribution of each putative donor genome is shown with different colors. In red, allelic variants of *TUBB* 4-1 and *TUBB* 1-1. Numbers on the chromosome diagrams indicate TBP amplicon length. **(B)** Sequence alignment of the β-tubulin *TUBB* 29-1 and *TUBB* 13-1 of *L. minor* 8627 with their L minor 5500 ortholog (contig Lm5500-3435), showing intron length variation due to simple sequence repeats. The *TUBB* 13-1 open reading frame is used for intron localization.

Allelic TBP variants were only found for locus *TUBB* 13-1 and its ortholog in *L. minor* 5500. A comparison of the 1st intron TBP profile of *L. minor* 8627 with those of clones 9495 and 9434, taken as representatives for *L. minor* SC I and *L. turionifera*, respectively, showed that it was an almost perfect merge of the other two (Figure 5A), strongly supporting the hypothesis of interspecific hybridization. The subset of TBP amplicons mapping to *L. minor* 8627 chromosomes 23, 29, and 39 (subgenome B) was present in *L. minor* 9495, while markers of the complementary subset, chromosomes 11, 13, and 26 (subgenome A), were found in *L. turionifera*. The CE-TBP peak of 598 bp, which is monomorphic between the two subgenomes and could be assigned either to *TUBB* 4-1 or *TUBB* 1-1 (Table 4), was present in both putative parental genomes, indicated in the diagram near each TBP profile as AA (*L. turionifera*) and BB (*L. minor*-SC II). Peaks of 364 and 385 bp in *L. minor* 8627 and 367 bp in *L. minor* 9495 were interpreted as allelic variants of *TUBB* 29-1 but could also be assigned to the homoeologous locus *TUBB* 13-1. In fact, their highly similar intron sequence hosts a microsatellite sequence based on CTT tandem repeats as shown in the sequence alignment in Figure 5B. This did not allow the precise assignment of these TBP amplicons to one or the other locus. The 2nd intron TBP profile comparison was also in agreement with the above-mentioned conclusion (not shown).

Since the sequence of the *atpF*-*atpH* plastid marker of *L. minor* 8627 matched those of *L. minor*/*L. japonica* rather than *L. turionifera*, *L. minor* was indicated as the maternal parent in the chromosome diagram. However, TBP analysis did not allow to conclude if *L. minor* 8627 could be an allotetraploid (AABB) or a homoploid hybrid (AB).

This result raised the question if the hybrid origin of *L. minor* 8627 from *L. turionifera* × *L. minor* breeding may also apply to all *L. minor*-SC II clones. Therefore, we used the TBP 1st intron profile of *L. minor* 8627 as a reference for the alignment of the TBP profiles of the 42 clones forming the *L. minor* cluster.

The raw numerical output of CE-TBP, indicating peak size and height, was converted into the simplified printout shown in Supplementary Table 4, in which each row corresponds to an accession, and each column corresponds to a specific amplicon size, whose presence is indicated by a symbol. Each amplicon was assigned to its putative locus based on fragment length calculation as reported in Table 4. Allelic length variants found among clones were tentatively assigned to the most probable locus by their length, also considering that one- to three-nucleotide-length variations were most likely due to the variable lengths of SSR that were present within intron sequences. Such SSR may produce a high number of allelic variants as in the case of *TUBB* 13-1 and *TUBB* 29-1, which showed a wide range of amplicons from 358 to 416 nucleotides in length, scattered by three nucleotides. Clones belonging to *L. minor*-SC II (excluding the *L. turionifera* subset) were characterized by a higher average number of amplicons than those of *L. minor*-SC I (10–13 vs. six to nine amplicons; last column in Supplementary Table 4). In fact, all clones showed amplicons corresponding to each of the β-tubulin loci of clone 8627 in accordance to their possible hybrid origin: those mapping at chromosomes 11 and 26 were shared with the three *L. turionifera* clones, 9434, 8693, and BOG0006, and those mapping at chromosomes 23 and 39 were shared with *L. minor*-SC I. Amplicons corresponding to loci on chromosomes 1 and 4, 13 and 29, could not be clearly assigned since they were either monomorphic across the three groups or highly polymorphic with overlapping length alleles.

Although not conclusive, these findings support the hypothesis that, similarly to clone 8627, all the clones in *L. minor*-SC II could be the result of interspecific hybridization between *L. turionifera* and *L. minor*.

### Single Tubulin Amplification

**FIGURE 6:**
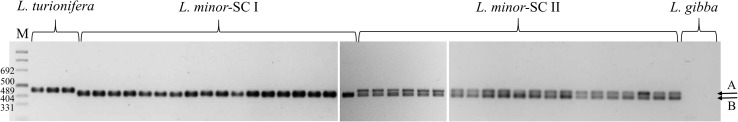
PCR amplification of all clones of the *Lemna minor* group with primers *TUBB* 23-11-1. From left to right: *Lemna turionifera*: 9434, BOG0006, 9530; *L. minor*-SC I: LM0010, LM0011, 8744, 5500, 7766, 9536, 8623, 7753, 7194, 8292, 7022, 9495, 9252, 9441, KJA0013, LM0008, 7210, 9533; *L. minor*-SC II: 8627, 8676, 8695, 9017, 9257, 9283, LM0005, LM0004, 9436b, 9580, LM0006, 9481, LM0007, 9437, KJA0017, KJA0016, 9355, 9440, 8389, 8625; *Lemna gibba*: 7796 and 7742a. The A and B arrows indicate the *L. turionifera* and *L. minor* bands, respectively. A molecular weight size marker (M) is reported on the left.

In order to exclude that peak attribution to β-tubulin loci exclusively based on amplicon length was fortuitous, we designed a primer pair specific for the exon–intron borders of the two putative β-tubulin homoeologs *TUBB* 11-1 and *TUBB* 23-1 of *L. minor* 8627, producing amplified fragments of 463 and 436 nucleotides, respectively. As shown in Figure 6, PCR amplification of all clones of the *L. minor* cluster revealed the presence of two distinct amplicons of expected length. The larger in size (amplicon A) was observed in the three investigated clones of *L. turionifera*, whereas the lower in size (amplicon B) was detected in all *L. minor*-SC I clones. As expected from their possible hybrid nature, all *L. minor*-SC II clones possessed both amplicons visible as a doublet in Figure 6. No amplification was found in two *L. gibba* clones, used as a control, suggesting a lesser degree of homology in the primer region. The amplification products of the three *L. turionifera* clones and three clones of both *L. minor*-SC II (9017, 9481, and 9530) and *L. minor*-SC I (7753, KJA0013, and 7022) were sequenced. Sequence analysis confirmed that amplicon A in the putative hybrids and that in *L. turionifera* have the same sequence of *TUBB* 11-1 of *L. minor* 8627 (overall identity 99% identity), while amplicon B was identical in sequence to those of *L. minor* SC I clones, to *L. minor* 8627 *TUBB* 23-1, and to its ortholog in *L. minor* 5500. Tubulin intron sequences are reported in the Supplementary Data text file.

### AFLP Analysis

To further confirm the hybrid nature of the *L. minor*-SC II group, an AFLP approach was used in order to provide genetic information at a genome-wide level rather than limited to a single gene family. Assuming that the *L. minor*-SC II group originated from hybridization between *L. minor*-SC I and *L. turionifera*, one would expect to detect a greater number of AFLP markers in the *L. minor*-SC II group when compared to the others and a low level of genetic diversity between the *L. minor*-SC II group and their putative parents.

Ten AFLP PCs were used to analyze the genomic DNA of the nine *Lemna* clones selected for sequencing of the *TUBB* 11-1/*TUBB* 23-1 loci and two *L. gibba* clones (7796 and 7742a). An average of 162 markers/PC was detected (Table 5). The estimated percentage of polymorphism of 99.98 highlighted the efficiency of the selected PCs for the fingerprinting of the selected group of species.

**TABLE 5:** Amplification and polymorphism information of 10 amplified fragment length polymorphism primer pairs.

	**TNM**	**NMM**	**PP**	**MNDL**			
				***L. turionifera***	***L. minor-*SC I**	***L. minor-*SC II**	***L. gibba***
PC47	177	4		73	66	92	50
PC48	145	3		46	46	70	44
PC49	101	5		37	42	54	31
PC50	206	1		71	73	99	69
PC51	186	2		61	58	85	58
PC52	176	0		62	57	82	58
PC53	88	6		29	34	39	51
PC54	154	3		47	60	71	57
PC55	161	10		72	83	83	64
PC56	225	6		101	86	119	85
Total	1,619	40	100	600	604	794	564
Mean	162	4		60	60	79	56
			N. fpm	44	26	8	260

*TNM, total number of markers; NMM, number of monomorphic markers; PP, percentage of polymorphism; MNDL, mean number of detected loci; N. fpm, number of fixed private markers.*

In agreement with our hypothesis, referring to the mean number of detected loci for each group of clones (Table 5), the AFLP output revealed an increase of 31, 32, and 41% in the *L. minor*-SC II group when compared to the *L. minor*-SC I, *L. turionifera*, and *L. gibba* groups, respectively. In addition, the number of fixed private markers calculated for each group (Table 5) strongly supports the presence of a hybrid profile for the *L. minor*-SC II group. In this group, the limited number of exclusive markers recorded, only eight, is due to the high percentage of markers mutually shared with the groups *L. minor*-SC I and *L. turionifera*. Conversely, the highest number of exclusive markers was detected in the *L. gibba* group, as expected.

The estimation of the Dice’s pairwise genetic distance among groups provided a further element of comparison. As reported in Table 6, the highest average values of genetic similarity were recorded comparing *L. minor*-SC II to both *L. minor*-SC I (0.5867) and *L. turionifera* (0.6568) groups, suggesting the presence of shared AFLP patterns. These data further corroborate the hypothesis of a hybrid origin for the *L. minor*-SC II group, which exhibits AFLP patterns sharing markers almost exclusively with the aforementioned groups. Conversely, the lowest values were estimated by comparing *L. gibba* to the other species.

**TABLE 6:** Mean Dice’s pairwise genetic distances estimated by the comparison of the four groups of clones.

	***L. turionifera***	***L. minor-*SC I**	***L. minor-*SC II**	***L. gibba***
*L. turionifera*	–			
*L. minor-*SC I	0.2904 ± 0.015	–		
*L. minor-*SC II	0.6568 ± 0.032	0.5867 ± 0.0401	–	
*L. gibba*	0.2243 ± 0.003	0.2547 ± 0.0165	0.248 ± 0.012	–

All our data are in perfect agreement with the conclusion that all clones in *L. minor*-SC II, also including *L. japonica*, are interspecific hybrids belonging to the larger group of *L. minor sensu lato*, while only clones in *L. minor*-SC I can be considered as *L. minor sensu stricto*. Re-classified and newly classified clones in stock collections were therefore renamed according to this conclusion.

## Discussion

### Species Delimitation by TBP

The successful testing of the molecular marker TBP for its capability of fingerprinting duckweed DNA for species discrimination was reported in the present study, conducted on a quite large number of duckweed clones of all species of the three genera *Spirodela*, *Landoltia*, and *Lemna.*

Tubulin-based polymorphism results, extensively validated by *atpF-atpH* barcoding, supported the classification of some new clones and the reclassification of some others from existing clone collections. Most species (10 out of 15) could be easily identified “at a glance” upon separation of PCR fragments on agarose gel, including *L. gibba*, easily mistaken for *L. minor* when gibbosity is not developed (De Lange and Westinga, 1979), or the invasive species *L. minuta* from the European native *L. minor* (Ceschin et al., 2016b). The use of a second primer pair (2nd intron TBP) provided an almost clear delineation of all species by cluster analysis, which was consistent with the morphological classification of clones and accepted relationships among closely related species as determined by AFLP fingerprinting or barcoding by sequence markers (Bog et al., 2010; Borisjuk et al., 2015; Tippery et al., 2015). Section delimitation was clear with the exception of *Lemna* that does not form a supported cluster and could not be separated from the other sections. Relationships between species within this section and between sections showed low bootstrap values, which were likely due to the limited number of shared markers and to the high number of intraspecific allelic variants.

### Interspecific Hybrids

The main problem in species delimitation is that TBP, as is the case with plastid markers, also looks unable to distinguish clones of *L. minor* from *L. japonica* which are intermingled in the same cluster, unless a different paradigm is applied, namely, that the separation of *L. minor* s.l. in two subclusters reflects the actual separation of the two species. Such separation has also supported the evidence that *Lemna minor-*SC I is characterized by a reduced number of TBP and AFLP loci per clone and by homozygosis of the B allele at the *TUBB* 23-1/11-1 locus. More easily identified by morphological traits and almost homogeneous, this group likely corresponds to *L. minor sensu stricto.* In fact, just one spurious clone, *L. japonica* 9252, here reclassified as *L. minor* by morphology, was present in this group.

Conversely, all clones in *L. minor-*SC II, despite their mixed classification as *L. japonica* or *L. minor*, display a higher number of TBP and AFLP loci, sharing approximately half of them with both *L. minor*-SC-I and *L. turionifera*, and are also heterozygous at the *TUBB* 23-1/11-1 locus. Sequence analysis confirmed that the two *TUBB* 23-1/11-1 alleles originated from *L. minor* (allele B) and *L. turionifera* (allele A), respectively. The *L. turionifera* group, branching from cluster SC II, possesses TBP alleles that are not found in any of the SC-I clones (Supplementary Table 4 and Figure 5) and is homozygous for the A allele at the *TUBB* 23-1/11-1 locus (Figure 6).

All these data are in agreement with the interpretation that clones in *L. minor-*SC II (*L. minor sensu lato*) are interspecific hybrids of *L. minor* × *L. turionifera*, thus representing a genetically different population from *L. minor sensu stricto*. If interspecific hybridization is an unlike occurrence in plants rarely flowering as duckweeds, such an event has a high chance of originating a new species, thanks to the rapid fixation of the newly generated chromosome set by fast vegetative propagation.

This finding also poses a question about the true identity of *L. japonica.* A possible interesting explanation is that this species, whose discrimination from *L. minor* is known to be particularly challenging by morphology and even by plastid barcoding, is an interspecific hybrid coinciding with the SC II group. This was, in fact, the original suspect of E. Landolt, who first described *L. japonica* as a species in 1980, suggesting its possible origin through hybridization of *L. minor* and *L. turionifera* on the basis of some intermediate morphologic characteristics (Landolt, 1980). However, attempts to cross *L. minor* and *L. turionifera* have never been successful (Landolt, 1986). Later, the hybrid hypothesis found contrasting supports from allozyme studies (Hirahaya and Kadono, 1995; Les et al., 2002), leaving this as an open question. However, such interpretation could explain the variable and overlapping morphological traits of clones in SC-II, some of which have been alternatively classified as *L. minor* or *L. japonica* and also as *L. turionifera* (clone 9530) by Landolt himself. For instance, clone 8767, recorded as *L. minor*, was lately reclassified by Landolt into *L. japonica* (personal communication to KA). Therefore, it should not be surprising that many clones in this group could have been mistakenly classified as *L. minor*, including the fully sequenced clone 8627, and should now be re-classified as *L. japonica*. According to the hybrid hypothesis, the maternal inheritance of plastids clearly explains the impossibility to discriminate *L. minor* from *L. japonica* by plastid markers (Borisjuk et al., 2015), while nuclear DNA investigation by AFLP (Bog et al., 2010; present paper) and genotyping by sequencing (Bog et al., 2020b) provided separation for the two species.

The karyotype of such hybrids remains an open question, which cannot be answered by TBP analysis. Although codominant, this marker cannot distinguish between an interspecific homoploid hybrid and an allotetraploid, unless more than two alleles are identified for the same locus within one clone.

Whole genome duplication by unreduced gamete production is a quite common consequence of interspecific hybridization in plants and the main mechanism to produce fertile hybrids, eventually leading to sympatric speciation (Rodionov et al., 2019). However, the preferred vegetative propagation of duckweeds makes fertility not a compelling requirement for speciation. By citing Elias Landolt on the subject of interspecific *Lemna* hybrids (1975): “if hybrids occur, they might become stabilized through vegetative reproduction and contribute in this way to some intergradation between certain species.” Whether *L. japonica* is sterile is not proven, but while flowering is known to occur rarely in both *L. minor* and *L. turionifera* and also in *L. japonica*, fruit setting was never observed in the last species (Bog et al., 2020a), although further investigation is required. Clone sterility would speak in favor of homoploidy of the hybrid.

An alternative possibility is that SC II is a heterogeneous cluster, likely characterized by different karyotypes, and the species described by Landolt as *L. japonica* corresponds to a subpopulation of such hybrids. In fact, it is possible that an original hybridization event was followed by different genomic rearrangements/polyploidization events that became stabilized in different lineages, possibly followed by further crosses or backcrosses, thus originating a reticulate evolution network quite complex to unravel. The existence of more than one original hybridization events, although unlikely, is also conceivable. Although conducted on a limited number of clones, our AFLP data using 10 primer combinations also revealed heterogeneity in the number of markers among SC II clones (not shown), which warrants further investigation.

Further genetic analyses are therefore needed to definitely characterize the genetic diversity and the population structure of the complex *L. minor/L. japonica* group and answer the open questions that have arisen from this work. Karyotyping analysis, in combination with whole genome sequencing of *L. turionifera* and other clones of *L. minor-*SC II, including *L. japonica*, could provide a conclusive response about the identity of *L. japonica*, thus confirming the discriminating power of TBP. More generally, the existence of interspecific hybrids in duckweeds has been postulated several times to explain some intermediate phenotypes found in the genus *Lemna* (Kandeler, 1975) or unresolved species delimitation by molecular markers, as in the case of *Wolffiella* (Bog et al., 2018).

### Clone Identification

Physiological and biochemical intraspecific variability, likely reflecting genetic diversity among clones of the same species, has been widely reported in duckweeds (Appenroth et al., 2013). However, most of the tested molecular markers seem to fail in providing an effective distinction at the clone level (Bog et al., 2019). One possible method capable of addressing this issue was recently presented by Chu et al. (2018), using the highly polymorphic regions of NBARC-related genes (nuclear-binding leucine-rich repeat protein) as markers. These authors were successful even in the case of *S. polyrhiza*, a species with very low genetic variations. However, this method, as well as inter-simple sequence repeat markers (ISSRs) or SSR markers, requires deep knowledge of the genome of each investigated species (Bog et al., 2019).

Thanks to the high resolution power of capillary electrophoresis, TBP was also able to highlight intraspecific variability in some species, mainly provided by the presence of SSR and short InDels in some β-tubulin introns. This was the case of the 18 *L. minor* clones in SC I, each showing a unique TBP profile. Analysis of higher numbers of clones is needed to ascertain the possibility of clone identification in other species, i.e., in largely heterogeneous species such as *L. gibba* or *L. aequinoctialis.*

## Conclusion

In conclusion, we believe that TBP analysis can be a simple and promising approach for species delimitation in the genus *Lemna* and to investigate similar cases of suspect hybridization in the duckweed family, thus contributing to elucidating duckweed speciation mechanisms. In contrast to barcoding using plastid *atpF-atpH* and *psbK-psbI* (Borisjuk et al., 2015), TBP provided a 100% score of correct identification until now, without any limit of PCR efficiency and without need for sequencing. The TBP marker can be considered a valid support to morphological analysis in many of those cases in which any previous classification is doubtful and could only be solved by further time-consuming investigations including flowering induction, turion forming ability, or other physiological or metabolic analysis (Les et al., 1997). It could also be adopted as a routine analysis to prevent clone exchange or cross-contaminations in large collections or in open-air cultivations, at least for the three investigated genera. A further application of the method to the remaining genera of Lemnaceae, *Wolffiella* and *Wolffia*, is ongoing.

## Data Availability Statement

The original contributions presented in the study are included in the article/Supplementary Material, further inquiries can be directed to the corresponding author/s.

## Author Contributions

LM, LB, and ML contributed to the conception and design of the study. ML, MB, and KA provided the clones. AG propagated plant material and extracted DNA. LB, ML, FG, and MB performed the experiments. LB, ML, and MB analyzed the TBP data and performed bioinformatic and statistical analysis. LM, LB, and KA collected and interpreted the data. DB and ML provided financial support. DB provided effective supervision. LM wrote the first draft of the manuscript. All authors contributed to the article and approved the submitted version.

## Conflict of Interest

The authors declare that the research was conducted in the absence of any commercial or financial relationships that could be construed as a potential conflict of interest.

## Footnotes

1 https://www.ncbi.nlm.nih.gov

2 http://www.bioinformatics.babraham.ac.uk/projects/trim_galore

## Supplementary Material

Supplementary Table 1 List of oligonucleotides used for amplified fragment length polymorphism analysis.

Supplementary Table 2 Capillary electrophoresis–tubulin-based polymorphism data (1st and 2nd intron) from *Spirodela polyrhiza*, *Spirodela intermedia*, and *Landoltia punctata* clones.

Supplementary Table 3 Capillary electrophoresis–tubulin-based polymorphism data (1st and 2nd intron) from 76 clones representative of the 12 *Lemna* species.

Supplementary Table 4 Schematic representation of the first intron capillary electrophoresis–tubulin-based polymorphism profiles of all clones of the *L. minor* cluster.

Supplementary Data Plastid barcoding sequences and genomic sequences of the *TUBB* 11-1 and *TUBB* 23-1 introns.
