# Supplementary material for: Duckweed Species Genotyping and Interspecific Hybrid Discovery by Tubulin-Based Polymorphism Fingerprinting
Source: Front Plant Sci. 2021 Mar 8;12:625670. doi: 10.3389/fpls.2021.625670 (PMC7982733; doi:10.3389/fpls.2021.625670)
Supplement: Supplementary Data — Plastid barcoding sequences and genomic sequences of the TUBB 11-1 and TUBB 23-1 introns. [file Data_Sheet_1.docx]

Supplementary Data

# Supplementary Data

**atpF-tpH cpDNA** **barcoding** **sequences**

>9260_atpH

ATTCTACAATAAAAAAGTAGGTCATAGACTTTTTGACTTAGACTTGCTTTTTGCTTCTTCGAATTATATCAACATTGCACTCTAACAATTACTTATTCGTTGAGAGAATACCTCCGGGAAGGACTGATTTTAGGATTAGTAATTAGCAGATCCTCTCGCTTTCTTCCTTCCCGTTTTTAGTTCTTAGTATAATGTAATGGAAAACTTGAGTATGCGTTGTAACGCAACAAACAAGGTATTTATCAATTGACAAAATAGTCAGGACCTAACCCAATAAGTATGTTCTTGTAATTGTAAACTTAAATTAGAATAAAATATTTAATTAGACTAAAATAAAATAATAAATACAAAATTTCTCAATTAAAAAATTTCATCTATTCCATTAAAAATCCCATAAAAAAAAAGAAATCAAACAAAGGGGGCGAAGTGATACAAAAAGAACTCTGTTCTTTGTTAGTCCTACCTATAAGAGGAGAGTAT

>9614_atpH

ATTCTAGAATCAAAAAAAGTACGTAATAGACTTTTTGACTTAGACTTGCTTTTTGCTTCTTCGAATTATATCAACATTGTACTCTAACAATTACTTATTCGTTGAGAGAATACCTCCGGGAAGGACTGATTTTAGGATTAGTAATTAGCAGATCCTCTCGCTTTCTTCCTTCCCGTTTTCAGTTCTTAGTATAATGTAATGCAAAATTTTTTAGAGTATGCGTTGTAACGCAATAAACAAGGTATTTACCAATTGACAAAATAGCCAGGACCTAACCCAATAAGTATGTTCTTGTAATTGTAAACTTTAATTAGAATTAAATAATAAATAAAAAAGTTCTCAATTAAATTAATTAATTTAATCTATTCCATTTTAAAATCCCATAAAAAAAAAGAAATCAAACAAAGGGGGCGAAGTAATACAAAAAGAACAGAACTCTGTTCTTTTTTAGTCCTATCTATAAGAGGAGAGTAT

>9583_atpH

ATTCTAGAATCAAAAAAAGTACGTAATAGACTTTTTGACTTAGACTTGCTTTTTGCTTCTTCGAATTATATCAACATTGTACTCTAACAATTACTTATTCGTTGAGAGAATACCTCCGGGAAGGACTGATTTTAGGATTAGTAATTAGCAGATCCTCTCGCTTTCTTCCTTCCCGTTTTCAGTTCTTAGTATAATGTAATGCAAAATTTTTTAGAGTATGCGTTGTAACGCAATAAACAAGGTATTTACCAATTGACAAAATAGCCAGGACCTAACCCAATAAGTATGTTCTTGTAATTGTAAACTTTAATTAGAATTAAATAATAAATAAAAAAGTTCTCAATTAAATTAATTAATTTAATCTATTCCATTTTAAAATCCCATAAAAAAAAAGAAATCAAACAAAGGGGGCGAAGTAATACAAAAAGAACAGAACTCTGTTCTTTTTTAGTCCTATCTATAAGAGGAGAGTAT

>9619_atpH

ATTCTAGAATCAAAAAAAGTACGTAATAGACTTTTTGACTTAGACTTGCTTTTTGCTTCTTCGAATTATATCAACATTGTACTCTAACAATTACTTATTCGTTGAGAGAATACCTCCGGGAAGGACTGATTTTAGGATTAGTAATTAGCAGATCCTCTCGCTTTCTTCCTTCCCGTTTTCAGTTCTTAGTATAATGTAATGCAAAATTTTTTAGAGTATGCGTTGTAACGCAATAAACAAGGTATTTACCAATTGACAAAATAGCCAGGACCTAACCCAATAAGTATGTTCTTGTAATTGTAAACTTTAATTAGAATTAAATAATAAATAAAAAAGTTCTCAATTAAATTAATTAATTTAATCTATTCCATTTTAAAATCCCATAAAAAAAAAGAAATCAAACAAAGGGGGCGAAGTAATACAAAAAGAACAGAACTCTGTTCTTTTTTAGTCCTATCTATAAGAGGAGAGTAT

>LM0009_atpH

ATTCTACAAAAAGAAAGTACTTTTTGACTTAGACTTGCTTTTTGCTTCTTCGAATTCTATCAACATTGCACTCTAACAATTACTTATTCGTTGAGAGAATACCTCCGGGAAGGACTGATTTTAGGATTAGTAATTAGCAGATCCTCTCGCTTTCTTCCTTCCCGTTTTTAGTTCTTAGTATAATGTAATGGAAAACTTTTTTGAGTATGCGTTGTAACGCAACAAACAAGGTATTTATTGACAAAATAGTCAGGCCCTAACCCAATAAGTATGCTCTTGTAATTGTAAACTTTAATTAGAATAAAATAATAAATAAAAAAGTTCTCAATTAAGTTAATTAATGTCATCTATTCCATTTTAAAATCCCATAAAAAAAAGAAATCAAAGAAAAGGGGCGAAGTAATACAAAAAGAACTCTGTTCTTTTTTAGTCCTATCTATAAGAGGAAAGTCT

>9434_atpH

ATTCTAGAATAAAAAAGTACGTAATAGACTTTTTGACTTAGACTTGCTTTTTGCTTCTTCGAATTATATCAAGATTGTACTCTAACAATTACTTATTCGTTGAGAGAATACCTCCGGGAAGGACTGATTTTAGGATTAGTAATTAGCAGATCCTCTCGCTTTCTTCCTTCCCGTTTTCAGTTCTTAGTATAATGTAATGCAAAACTTTTTTTGAGTATGCGTTGTAACGCAACAAACAAGGTATTTATCAATTGACAAAATAGCCAGGATCTAACCCAATAAGTATGTTCTTGTAATTATAAACTTTAATTAGAATTAAATAATAAATAAAAAAGTTCTCAATTAAATTAATTAATTAAATCTATTCCATTTAAAAATCCCATAAAAAAAAGAAATCAAACAAAGGGGGCGAAGTAATACAAAAAGAACTCTGTTCTTTTTTAGTCCTATCTATAAGAGGAGAGTAT

>7245_atpH

ATTTCTAGAATCAAAAAAAGTACGTAATAGACTTTTTGACTTAGACTTGCTTTTTGCTTCTTCGAATTATATCAACATTGTACTCTAACAATTACTTATTCGTTGAGAGAATACCTCCGGGAAGGACTGATTTTAGGATTAGTAATTAGCAGATCCTCTCGCTTTCTTCCTTCCCGTTTTCAGTTCTTAGTATAATGTAATGCAAAATTTTTTAGAGTATGCGTTGTAACGCAATAAACAAGGTATTTACCAATTGACAAAATAGCCAGGACCTAACCCAATAAGTATGTTCTTGTAATTGTAAACTTTAATTAGAATTTAATAATAAATAAAAAAGTTCTCAATTAAATTAATTAATTTAATCTATTCCATTTTAAAATCCCATAAAAAAAAAGAAATCAAACAAAGGGGGCGAAGTAATACAAAAAGAACAGAACTCTGTTCTTTTTTAGTCCTATCTATAAGAGGAGAGTAT

>9481_atpH

ATTCTAGAATAAAAAAGTACGTAATAGACTTTTTGACTTAGACTTGCTTTTTGCTTCTTCGAATTATATCAACATTGTACTCTAACAATTACTTATTCGTTGAGAGAATACCTCCGGGAAGGACTGATTTTAGGATTAGTAATTAGCAGATCCTCTCGCTTTCTTCCTTCCCGTTTTCAGTTCTTAGTATAATGTAATGCAAAACTTTTTTGAGTATGCGTTGTAACGCAATAAACAAGGTATTTACCAATTGACAAAATAGCCAGGACCTAACCCAATAAGTATGTTCTTGTAATTGTAAACTTTAATTAGAATTAAATAATAAATAAATAAAGTTCTCAATTAAATTAATTAATTTAATCTATTCCATTTTTAAATCCCATAAAAAAAAAAAAGAAATCAAACAAAGGGGGCGAAGTAATACAAAAGGAACTCTGTTCTTTTTTAGTCCTATCTATAAGAGGAGAGTATATGCAAA

>8676_atpH

AATTCTAGAATAAAAAAGTACGTAATAGACTTTTTGACTTAGACTTGCTTTTTGCTTCTTCGAATTATATCAACATTGTACTCTAACAATTACTTATTCGTTGAGAGAATACCTCCGGGAAGGACTGATTTTAGGATTAGTAATTAGCAGATCCTCTCGCTTTCTTCCTTCCCGTTTTCAGTTCTTAGTATAATGTAATGCAAAACTTTTTTGAGTATGCGTTGTAACGCAATAAACAAGGTATTTACCAATTGACAAAATAGCCAGGACCTAACCCAATAAGTATGTTCTTGTAATTGTAAACTTTAATTAGAATTAAATAATAAATAAATAAAGTTCTCAATTAAATTAATTAATTTAATCTATTCCATTTTTAAATCCCATAAAAAAAAAAAARAAATCAAACAAAGGGGGCGAAGTAATACAAAAGGAACTCTGTTCTTTTTTAGTCCTATCTATAAGAGGAGAGTAT

>LM0010_atpH

AAAAAAAAGTACGTAATAGACTTTTTGACTTAGACTTGCTTTTTGCTTCTTCGAATTATATCAACATTGTACTCTAACAATTACTTATTCGTTGAGAGAATACCTCCGGGAAGGACTGATTTTAGGATTAGTAATTAGCAGATCCTCTCGCTTTCTTCCTTCCCGTTTTCAGTTCTTAGTATAATGTAATGCAAAACTTTTTTGAGTATGCGTTGTAACGCAATAAACAAGGTATTTACCAATTGACAAAATAGCCAGGACCTAACCCAATAAGTATGTTCTTGTAATTGTAAACTTTAATTAGAATTAAATAATAAATAAATAAAGTTCTCAATTAAATTAATTAATTTAATCTATTCCATTTTTAAATCCCATAAAAAAAAAAAAGAAATCAAACAAAGGGGGCGAAGTAATACAAAAGGAACTCTGTTCTTTTTTAGTCCTATCTATAAAAGGAGAGTATA

>LM0011_atpH

ATTTCCTAGAATAAAAAAGTACGTAATAGACTTTTTGACTTAGACTTGCTTTTTGCTTCTTCGAATTATATCAACATTGTACTCTAACAATTACTTATTCGTTGAGAGAATACCTCCGGGAAGGACTGATTTTAGGATTAGTAATTAGCAGATCCTCTCGCTTTCTTCCTTCCCGTTTTCAGTTCTTAGTATAATGTAATGCAAAACTTTTTTGAGTATGCGTTGTAACGCAATAAACAAGGTATTTACCAATTGACAAAATAGCCAGGACCTAACCCAATAAGTATGTTCTTGTAATTGTAAACTTTAATTAGAATTAAATAATAAATAAATAAAGTTCTCAATTAAATTAATTAATTTAATCTATTCCATTTTTAAATCCCATAAAAAAAAAAAAGAAATCAAACAAAGGGGGNGAAGTAATACAANAGGAACTCTGTTCTTTTTTAGTCCTATCTATAAGAGGAGAGTA

>8627_atpH

ATTCTAGAATAAAAAAGTACGTAATAGACTTTTTGACTTAGACTTGCTTTTTGCTTCTTCGAATTATATCAACATTGTACTCTAACAATTACTTATTCGTTGAGAGAATACCTCCGGGAAGGACTGATTTTAGGATTAGTAATTAGCAGATCCTCTCGCTTTCTTCCTTCCCGTTTTCAGTTCTTAGTATAATGTAATGCAAAACTTTTTTGAGTATGCGTTGTAACGCAATAAACAAGGTATTTACCAATTGACAAAATAGCCAGGACCTAACCCAATAAGTATGTTCTTGTAATTGTAAACTTTAATTAGAATTAAATAATAAATAAATAAAGTTCTCAATTAAATTAATTAATTTAATCTATTCCATTTTTAAATCCCATAAAAAAAAAAAAGAAATCAAACAAAGGGGGCGAAGTAATACAAAAGGAACTCTGTTCTTTTTTAGTCCTATCTATAAGAGGAGAGTA

>8744_atpH

ATTCTAGAATAAAAAAGTACGTAATAGACTTTTTGACTTAGACTTGCTTTTTGCTTCTTCGAATTATATCAACATTGTACTCTAACAATTACTTATTCGTTGAGAGAATACCTCCGGGAAGGACTGATTTTAGGATTAGTAATTAGCAGATCCTCTCGCTTTCTTCCTTCCCGTTTTCAGTTCTTAGTATAATGTAATGCAAAACTTTTTTGAGTATGCGTTGTAACGCAATAAACAAGGTATTTACCAATTGACAAAATAGCCAGGACCTAACCCAATAAGTATGTTCTTGTAATTGTAAACTTTAATTAGAATTAAATAATAAATAAATAAAGTTCTCAATTAAATTAATTAATTTAATCTATTCCATTTTTAAATCCCATAAAAAAAAAAAAGAAATCAAACAAAGGGGGCGAAGTAATACAAAAGGAACTCTGTTCTTTTTTAGTCCTATCTATAAGAGGAGAGTA

>7269_atpH

TTAgCACTTTTaTTTGCGaATCcATTTGTTTAATTCTAGAATCAAAAAAAGTACGTAATAgACttTTtGACTTAGACTTGCTTTTTGCTTCTTCGAATTATATCaacATTGTACTCTAACAATTACTTATTCGTTGAGAGAATACCTCCGGGAAGGACTGATTTTAGGATTAGTAATTAgCAGATCCTCTCGCTTTCTTCCTTCCCGTTTTCAGTTCTTAGTATAATGTAATGCAAAATTTTTTAGAGTATGCGTTGTAACGCAATAAACAAGGTATTTACCAATTGACAAAATAGCCAGGACCTAACCCAATAAGTATGTTCTTGTAATTGTAAACTTTAATTAGAATTAAATAATAAATAAAAAAGTTCTCAATTAAATTCATTAATTTCATCTATTCCATTTTAAAATCCCATAAAAAAAAGAAATCAAACAAAGGGGGCGAAGTAATACAAAAAGAACTCTGTTCTTTTTTAGTCCTATCTATAAGAGGAGAGTATATGCAAAATGTAACTGATTCTTTTGTTTCCTTGGGCCACTGGCCATccgccggtggttttgggtttaataccgatattttagcaacaaat

>8473_atpH

TTAGCaCTTTTATTTGCGAaTCCaTTTGTTTAaTTCTACAAAAAGAAAGTACTTTTTGACTTcGACTTGATTTTTGCTTCTTCgAATTCTATCAACATTGCACTCTAACAAtTACTTATTCGTTGAGAGAATACCTCCGGGAAGGACTGATTTTAGGATTAGTAATTAGCAGATCCTCtCgcTTTCTTCCTTCCCGTTTTTAGTTCTTAgTATAATGTAATGGAAAAcTTTTTTGAGtATGCGTTGTAACGCAACAAAcAAGGtATTtATTGATAAAATAtcAGGCcCTAACCCAATAAGTATGCTcTTGtAATTGTAAACTTTAATTAGAATAAAATAATAAATAAAA

>8693_atpH

TTAGCACTTTTATTTGCGAATCCATTTGTTTAATTCTAGAATAAAAAAGTACGTAATAGACTTTTTGACTTAGACTTGCTTTTTGCTTCTTCGAATTATATCAAGATTGTACTCTAACAATTACTTATTCGTTGAGAGAATACCTCCGGGAAGGACTGATTTTAGGATTAGTAATTANNNNN

>8831_atpH

TtAgCaCTTTTATTTGCGAATCCATTTGTTTAATTCTACAATAAAAAAGTAGGTCAtAGaCtTtttGAcTTAgACTTGCTTTTTGCTTCTTCGAATTATATCAACATTgCACTCTAACaATTACTTATTCGTtGAGAGAATACCTCCGGGAAGGACTGATTTTAGGATTAGTAATTAgCAGATCCTCTCGCTTTCTTCCTTCCCGTTTTTAGTTCTTAGTATAATGTAATGGAAAACTTGAGTATGCGTTGTAACGcAAcAAACAAGgtatttatcaattgacaaaatagtcaggacctaacccaataa

>9020_atpH

TTAgCaCTTTTATTTGCGAATCCATTTGTTTAATTCTACAAAAAAGAAAGTACGTAATAGACTTTTtGACTTAGACTTGCTTTTTGATTCTTCGAATTCTATCAACaTTGCACTCTAACAATTACTTATTCGTTGAGAGAATACCTCCGGGAAGGACTGATTTTAGGATTAGTAATTAGCAGATCCTCTCGCTTTCTTCCTTCCCGTTTTTAGTTCTTAGTATAATGTAATGGAAAACTTTTTTGAGTATGCGTTGTAACGCAACAAATAAGGTATTTATTGACAAAACAGTCAGGACCTAACCCAATAAGTATGCTCTTGTAATTGTAAACTTTAATTAGAATAAAATAATAAATAAAAAAGTTCTCAATTAAGTTAATTAATGTCATCTATTCCATTTTAAAATCCCATAAAAAAAAAAAgAAATCAAAGAAAAGGGGCGAAgTAATACAAAAAGAACTCTGTTCTTTGTTAGTCCTATCTATAAgAGGAAAGtAtATGCAAAATGTAACTGATTCTTTTGTTTCCTTGGGCCACTGGCCGTCTGCCGGGGGTTTTGGGTTTAATACCgATaTTTtAgcaaCAAAT

>9024_atpH

TTaGCACTTTTATTTGCGAATCCATTTGTTTAATTCTACAAAaAAGAAAGTACGTAATAgACTtttTGACtTAGACTTGCTTTTTGATTCTTCGAATTCTATCaacaTTGCACTCTAACAATTACTTATTCGTTGAGAGAatACCtCCgGgAAGGAcTGATTTTAGGAttAgTAATtAgcA

>9229_atpH

TTAGCACTTTTATTTGCGAaTCCATTTGTTTAATTCTACAAAAGTAGGTCATAGACTTTtTGACTTAGACTTGCTTTTTGCTTCTTCGAATTATATCAACATTGCACTCTAACAATTACTTATTCGTTGAGAGAATACCTCCGGGAAGGACTGATTTTAGGATTAGTAATTAGCAGATCCTCTCGCTTTCTTCCTTCCCGTTTTTAGTTCTTAGTATAATGTAATGGAAAACTTGAGTATGCGTTGTAACGCAACAAACAAGGTATTTATCAATTGACAAAATAGTCAGGACCTAACCCAATAAGTATGTTCTTGTAATTGTAAACTTAAATTAGAATAAAATATTTAATTAGACTAAAATAATAAATACAAAATTTCTCAATTAAAAAATTTCATCTATTCCATTAAAAATCCCATAAAAAAAAGAAATCAAACAAAGGGGGCGAAGTGATACAAAAAGAACTCTGTTCTTTGTTAGTCCTACCTATAAGAGGAGAGTATATGCAAAATGTAACTGATTCTTTTGTTTCCTTGGGCCACTGGCCATACGCCGGGGGTTTTGGGTTTAATACCGATATTTTAGCAACAAATCCCAATAAAATCTAAGTGTAGTACTTGAGTGTAATAATTTATTTTGGAAAAGGGAGTGTGTGCCGAAGTATTAG

>9530_atpH

TTAgCACTTTTATTTGCGAATCCATTTGTTTaATTCTAGAATAAAAAAGTACGTAATAGAcTttttGActTAgACTTGCTTTTTGCTTCTTCGAATTATATCaaCaTTGtACTCTAACAATTACTTATTCGTTGAGAGAATACCTCCGGGAAGGACTGATTTTAGGATTAGTAATTAgcagATCCTCTCGCTTTCTTCCTTCCCGTTTTCAGTTCTTAGTATAATGTAATGCAAAACTTTTTTGAGTATgCGTTGTAACGCaATAAACA

>BOG0001_atpH

TTaGCACTTTTATTTGCGAATCCATTTGTTTAATTCTACAAAAAAGAAAGTACTTTTTGACTTAgACTTGCTTTTTGCTTCTTCGAATTCTATCAACATTGCaCTCTAACAATTACTTATTCGTTGAGAGAATACCTCCGGGAAGGACTGATTTTAGGATTAGTAATTAGCAGATCCTCTCGCTTTCTTCCTTCCCGTTTTTAGTTCTTAGTATAATGTAAGGGAAAACTTTTTTGAGTATGCGTTGTAACGCAACAAACAAGGTATTTATTGACAAAATAGTCAGGCCCTAACCCAATAAGTATGCTCTTGTAATTGTAAACTTTAATTAGAATAAAATAATAAATAAAAAAGTTCTCAATTAAGTTAATTAATGTCATCTATTCTATTTTAAAATCCCATAAAAAAAAAAAAAaAgAAAtCAAAGAAAAGGGGcgAAGtAat

>BOG0006_atpH

TTAGCaCTTTTATTTGCGaATCCATTTGTTTAATTCTAGAATAAAAAAGTACGTAATAgACTTTTTGACTTAGACTTGCTTTTTGCTTCTTCGAATTATATCAAGATTGTACTCTAACAATTACTTATTCGTTGAGAGAATACCTCCGGGAAGGACTGATTTTAGGATTAGTAATTAGCAGATCCTCTCGCTTTCTTCCTTCCCGTTTTCAGTTCTTAGTATAATGTAATGCAAAACTTTTTTTGAGTATGCGTTGTAACGCAACAAACAAGGTATTTATCAATTGACAAAATAGCCAGGATCTAACCCAATAAGTATGTTCTTGTAATTATAAACTTTAATTAGAATTAAATAATAAATAAAAAAGTTCTCAATTAAATTAATTAATTAAATCTATTCCATTTAAAAATCCCATAAAAAAAAGAAATCAAACAAAGGGGGCGAAGTAATACAAAAAGAACTCTGTTCTTTTTTAGTCCTATCTATAAGAGGAGAGTATATGCAAAATGTAACTGATTCTTTTGTTTCCTTGGGCCACTGGCCATCCGCCGGCGGTTTTGGGTTTAATACCGAtATTTTAGCAACAAAT

>9017_atpH

TTaGCaCTTTTATTTGCGAaTCCaTTTGTTTaATTCTAGAATAAAAAAGTACGTAATAgACTTTTTGACTTAGACTTGCTTTTTGCTTCTTCGAATTATATCaACATTGtACTCTAACAATTACTTATTCGTTGAGAGAATACCTCCGGGAAGGACTGATTTTAGGATTAGTAATTAGCAGATCCTCTCGCTTTCTTCCTTCCCGTTTTCAGTTCTTAGTATAATGTAATGCAAAACTTTTTTGAGTATGCGTTGTAACGCAATAAACAAGGTATTTACCAATTGACAAAATAGCCAGGACCTAACCCAATAAGTATGTTCTTGTAATTGTAAACTTTAATTAGAATTAAATAATAAATAAATAAAGTTCTCAATTAAATTAATTAATTTAATCTATTCCATTTTTAAATCCCATAAAAAAAAAAAAAGAAAtcAAACAAaGGGGGCGAAGtAATAC

**Genomic sequences of the *TUBB* 11-1 and *TUBB* 23-1 introns**

>Contig_minor_SC1_7022

GATATAGTTATGAATGTCTTTGTGTATTTTTAGGGTTTGATATTTCTGATTACCGGATCTGTACCACAAAGCCTTGCAATATTCTCATCCTTGCCGATTTGTGCCTGTCTTTGGGTTTTGATATTCAGATCTACATCGTGCTCTTTATGTTTATAAGAAACATGGATCCGATATTGTTTTCTCCTGTTTCGTTTTACCTTATTGCAGTGACAATGTCTTTCTGATCAAGGATCTATTTGCGTCATTTTTGTGTGCTGAGATTTACATTCCTCATGATCTATTTGTGTCATATGATCCAACCGAATATTCTCATACTCTACGAAATTCAGTGTTTCTGTGTCAAACTCTTTCGTCTCATATTGGTTTACTTCCCCCGTCATTTACTTGCAGGATTCCA

>Contig_minor_SC1_7753

TATAGTTATGAATGTCTTTGTGTATTTTTAGGGTTTGATATGTCTGATCACCGGATCTGTACCACAAAGCCTTGCAATATTCTCATCCTTGCCGATTTGTGCCTGTCTTTGGGTTTTGATATTCAGATCTACATCGTGTTCTTTATGTTTAAAAGAAACAGGGATCCGATATTGTTTTCTCCTGTTTCGTTTTACCTTATTGCTGCTGACAATGTCTTTCTGATCAAGGATATATTTGCGTCATTTTTGTGTGCTGAGATTTACATTCCTCAGTGATCTATTTGTGTCATATAATCCAACCGAATATTCTCATACTCTACGAAATTCAGTGTTTCTGTGTTAAACTCTTTCGTCTCATATTGGTTTACTTCCCCCGTCATTTACTTGCAGGATTCCA

>Contig_Turion8693

CAGGGTATGCGATCTATTCTTTTCAATGTCCTTCATAGATAAAATTATGAAAGTGTTTGGGCAGTGTTAGGGTCTGATTCGTTTGGTTTCGGAATCTGTACCTCAAAGCCTTACAGTATTCACTCTTGCCGATTTTTTCCTGTGTTTGTATGTTCACATTCGCTGTAGATCTAGATCGTGTTATTTAAGATAAGAGAAAAATAGATCCATTACTGTTGCCTTCTGTTTCGTATTTTTCGTGTTGCGGTGACAATGAACATACGATGTAACTCTAATCAAGTGTGATCTATTTGCATCCCTCACTATTTGATGAAACCCAGGCTGACATCCCTCAGGATCTGTTTGTTTCATTTGTTCCGATCAAAATTCTTCTACCCGATGCTTATATTTACTGGGTTTGCGTGCTAAAATTTCTCAGTCTCATTTTGGTTAGCTTCCCCCGTCATTTACTTGCAGG

>Contig_9017A

TTTCAGGGTATGCGATCTATTCTTTTCAATGTCCTTCATAGATAAAATTATGAAAGTGTTTGGGCAGTGTTAGGGTCTGATTCGTTTGGTTTCGGAATCTGTACCTCAAAGCCTTACAGTATTCACTCTTGCCGATTTTTTCCTGTGTTTGTATGTTCACATTCGCTGTAGATCTAGATCGTGTTATTTAAGATAAGAAAAAAATAGATCCATTACTGTTGCCTTCTGTTTCGTATTTTTCGTGTTGCGGTGACAATGAACATACGATGTAACTCTAATCAAGTGTGATCTATTTGCATCCCTCACTATTTGATGAAACCCAGGCTGACATCCCTCAGGATCTGTTTGTTTCATTTGTTCCGATCAAAATTCTTCTACCCGATCTTATATTTACTGGGTTTGCGTGCTAAAATTTCTCAGTCTCATTTTGGTTAGCTTCCCCCGTCATTTACTTGCAGG

>Contig_9017B

AGGGTATGCGATCTATTCTTTTCAATGTTGTTCATAGAATATAGTTATGAATGTCTTTGTGTATTTTTAGGGTTTGATATTTCTGATTACCGGATCTGTACCACAAAGCCTTGCAATATTCTCATCCTTGCCGATTTGTGCCTGTCTTTGGGTTTTGATATTCAGATCTACATCGTGCTCTTTATGTTTATAAGAAACATGGATCCGATATTGTTTTCTCCTGTTTCGTTTTACCTTATTGCAGTGACAATGTCTTTCTGATCAAGGATCTATTTGCGTCATTTTTGTGTGCTGAGATTTACATTCCTCATGATCTATTTGTGTCATATGATCCAACCGAATATTCTCATACTCTACGAAATTCAGTGTTTTCTGTGTCAAACTCTTTCGTCTCATATTGGTTTACTTCCCCCGTCATTTACTTGCAGGATTCCA

>Contig_Turion9434

CAGGGTATGCGATCTATTCTTTTCAATGTCCTTCATAGATAAAATTATGAAAGTGTTTGGGCAGTGTTAGGGTCTGATTCGTTTGGTTTCGGAATCTGTACCTCAAAGCCTTACAGTATTCACTCTTGCCGATTTTTTCCTGTGTTTGTATGTTCACATTCGCTGTAGATCTAGATCGTGTTATTTAAGATAAGAGAAAAATAGATCCATTACTGTTGCCTTCTGTTTCGTATTTTTCGTGTTGCGGTGACAATGAACATACGATGTAACTCTAATCAAGTGTGATCTATTTGCATCCCTCACTATTTGATGAAACCCAGGCTGACATCCCTCAGGATCTGTTTGTTTCATTTGTTCCGATCAAAATTCTTCTACCCGATCTTATATTTACTGGGTTTGCGTGCTAAAATTTCTCAGTCTCATTTTGGTTAGCTTCCCCCGTCATTTACTTGCAGG

>Contig_9481B

TTTCAGGGTATGCGATCTATTCTTTTCAATGTTGTTCATAGAATATAGTTATGAATGTCTTTGTGTATTTTTAGGGTTTGATATTTCTGATTACCGGATCTGTACCACAAAGCCTTGCAATATTCTCATCCTTGCCGATTTGTGCCTGTCTTTGGGTTTTGATATTCAGATCTACATCGTGCTCTTTATGTTTATAAGAAACATGGATCCGATATTGTTTTCTCCTGTTTCGTTTTACCTTATTGCAGTGACAATGTCTTTCTGATCAAGGATCTATTTGCGTCATTTTTGTGTGCTGAGATTTACATTCCTCATGATCTATTTGTGTCATATGATCCAACCGAATATTCTCATACTCTACGAAATTCAGTGTTTCTGTGTCAAACTCTTTCGTCTCATATTGGTTTACTTCCCCCGTCATTTACTTGCAGGATTCCA

>Contig_9530B

AGGGTATGCGATCTATTCTTTTCAATGTTGTTCATAGAATATAGTTATGAATGTCTTTGTGTATTTTTAGGGTTTGATATTTCTGATTACCGGATCTGTACCACAAAGCCTTGCAATATTCTCATCCTTGCCGATTTGTGCCTGTCTTTGGGTTTTGATATTCAGATCTACATCGTGCTCTTTATGTTTATAAGAAACATGGATCCGATATTGTTTTCTCCTGTTTCGTTTTACCTTATTGCAGTGACAATGTCTTTCTGATCAAGGATCTATTTGCGTCATTTTTGTGTGCTGAGATTTACATTCCTCATGATCTATTTGTGTCATATGATCCAACCGAATATTCTCATACTCTACGAAATTCAGTGTTTCTGTGTCAAACTCTTTCGTCTCTTATTGGTTTACTTCCCCCGTCATTTACTTGCAGGATTCCA

>Contig_TurionBOG0006

CGTCCTTATAGAAAAATTATGAAGTGTTTGGGCAGTGTTAGGGTCTGATTCGTTTGGTTTCGGAATCTGTACCTCAAAGCCTTACAGTATTCACTCTTGCCGATTTTTTCCTGTGTTTGTATGTTCACATTCGCTGTAGATCTAGATCGTGTTATTTAAGATAAGAGAAAAATAGATCCATTACTGTTGCCTTCTGTTTCGTATTTTTCGTGTTGCGGTGACAATGAACATACGATGTAACTCTAATCAAGTGTGATCTATTTGCATCCCTCACTATTTGATGAAACCCAGGCTGACATCCCTCAGGATCTGTTTGTTTCATTTGTTCCGATCAAAATTCTTCTACCCGATCTTATATTTACTGGGTTTGCTGTGCTAAAATTTCTCAGTCTCATTTTGGTTAGCTTCCCCCGTCATTTACTTGCAGG

>Contig_9530A

TTCAGGGTATGCGATCTATTCTTTTCAATGTCCTTCATAGATAAAATTATGAAAGTGTTTGGGCAGTGTTAGGGTCTGATTCGTTTGGTTTCGGAATCTGTACCTCAAAGCCTTACAGTATTCACTCTTGCCGATTTTTTCCTGTGTTTGTATGTTCACATTCGCTGTAGATCTAGATCGTGTTATTTAAGATAAGAGAAAAATAGATCCATTACTGTTGCCTTCTGTTTCGTATTTTTCGTGTTGCGGTGACAATGAACATACGATGTAACTCTAATCAAGTGTGATCTATTTGCATCCCTCACTATTTGATGAAACCCAGGCTGACATCCCTCAGGATCTGTTTGTTTCATTTGTTCCGATCAAAATTCTTCTACCCGATCTTATATTTACTGGGTTTGTGTGCTAAAATTTCTCAGTCTCATTTTGGTTAGCTTCCCCCGTCATTTACGTGCAGGATTCc

>Contig_9481A

TTCAGGGTATGCGATCTATTCTTTTCAATGTCCTTCATAGATAAAATTATGAAAGTGTTTGGGCAGTGTTAGGGTCTGATTCGTTTGGTTTCGGAATCTGTACCTCAAAGCCTCACAGTATTCACTCTTGCCGATTTTTTCCTGTGTTTGTATGTTCACATTCGCTGTAGATCTAGATCGTGTTATTTAAGATAAGAGAAAAATAGATCCATTACTGTTGCCTTCTGTTTCGTATTTTTCGTGTTGCGGTGACAATGAACATACGATGTAACTCTAATCAAGTGTGATCTATTTGCATCCCTCACTATTTGATGAAACCCAGGCTGACATCCCTCAGGATCTGTTTGTTTCATTTGTTCCGATCAAAATTCTTCTACCCGATCTTATATTTACTGGGTTTGCGTGCTAAAATTTCTCAGTCTCATTTTGGTCAGCTTCCCCCGTCATTTACTTGCAGGATTCC
